# Multiple phylogenetically-diverse, differentially-virulent *Burkholderia pseudomallei* isolated from a single soil sample collected in Thailand

**DOI:** 10.1371/journal.pntd.0010172

**Published:** 2022-02-10

**Authors:** Chandler Roe, Adam J. Vazquez, Paul D. Phillips, Chris J. Allender, Richard A. Bowen, Roxanne D. Nottingham, Adina Doyle, Gumphol Wongsuwan, Vanaporn Wuthiekanun, Direk Limmathurotsakul, Sharon Peacock, Paul Keim, Apichai Tuanyok, David M. Wagner, Jason W. Sahl

**Affiliations:** 1 The Pathogen and Microbiome Institute, Northern Arizona University, Flagstaff, Arizona, United States of America; 2 Department of Biological Sciences, Colorado State University, Ft. Collins, Colorado, United States of America; 3 Mahidol-Oxford Tropical Medicine Research Unit, Mahidol University, Bangkok, Thailand; 4 Department of Medicine, University of Cambridge, Cambridge, England; National University of Singapore, SINGAPORE

## Abstract

*Burkholderia pseudomallei* is a soil-dwelling bacterium endemic to Southeast Asia and northern Australia that causes the disease, melioidosis. Although the global genomic diversity of clinical *B*. *pseudomallei* isolates has been investigated, there is limited understanding of its genomic diversity across small geographic scales, especially in soil. In this study, we obtained 288 *B*. *pseudomallei* isolates from a single soil sample (~100g; intensive site 2, INT2) collected at a depth of 30cm from a site in Ubon Ratchathani Province, Thailand. We sequenced the genomes of 169 of these isolates that represent 7 distinct sequence types (STs), including a new ST (ST1820), based on multi-locus sequence typing (MLST) analysis. A core genome SNP phylogeny demonstrated that all identified STs share a recent common ancestor that diverged an estimated 796–1260 years ago. A pan-genomics analysis demonstrated recombination between clades and intra-MLST phylogenetic and gene differences. To identify potential differential virulence between STs, groups of BALB/c mice (5 mice/isolate) were challenged via subcutaneous injection (500 CFUs) with 30 INT2 isolates representing 5 different STs; over the 21-day experiment, eight isolates killed all mice, 2 isolates killed an intermediate number of mice (1–2), and 20 isolates killed no mice. Although the virulence results were largely stratified by ST, one virulent isolate and six attenuated isolates were from the same ST (ST1005), suggesting that variably conserved genomic regions may contribute to virulence. Genomes from the animal-challenged isolates were subjected to a bacterial genome-wide association study to identify genomic regions associated with differential virulence. One associated region is a unique variant of Hcp1, a component of the type VI secretion system, which may result in attenuation. The results of this study have implications for comprehensive sampling strategies, environmental exposure risk assessment, and understanding recombination and differential virulence in *B*. *pseudomallei*.

## Author summary

*Burkholderia pseudomallei* is the causative agent of melioidosis, a disease endemic to Southeast Asia and northern Australia. The evaluation of diversity within *B*. *pseudomallei* has largely been conducted utilizing clinical isolates despite almost all infections emerging from environmental exposure. In this study, we surveyed the genomic diversity of 169 isolates collected from a single soil sample in Ubon Ratchathani Province, Thailand. Seven different sequence types were identified, and substantial within-sequence-type gene diversity was observed. To test for differential virulence, 30 isolates were challenged in a mouse melioidosis model. A small number of isolates killed all mice, but most killed none, demonstrating the variable virulence potential of different *B*. *pseudomallei* isolates present in the single sample. A comparative genomics analysis identified multiple genes associated with virulence, including Hcp1, a component of the type VI secretion system and a known virulence factor. The results of this study have implications for the comprehensive environmental surveillance, environmental exposure, and differential virulence of *B*. *pseudomallei*.

## Introduction

*Burkholderia pseudomallei* is a soil-dwelling bacterium endemic to Southeast Asia and northern Australia where it is the causative agent of melioidosis, a potentially fatal disease in humans [1]. In Thailand, *B*. *pseudomallei* causes 19% of community-acquired bacteremia [2] and in northern Australia, melioidosis is the most common cause of fatal bacteremic pneumonia [3]. Agricultural workers are among the highest at-risk population to develop infection due to repeated exposure to *B*. *pseudomallei*, primarily due to direct contact with compromised skin [4]. Melioidosis can be difficult to treat with antibiotics [5] and has been associated with a mortality rate as high as 50% in symptomatic individuals [6]. The most probable route of infection occurs through percutaneous inoculation [7], although inhalation and ingestion are also important [8,9].

Most genome sequencing of *B*. *pseudomallei* has focused on clinical isolates, although sequencing and analysis of environmental isolates has been performed, including recent studies focused on novel diversity in the Caribbean [10,11]. Other studies have investigated the microdiversity of *B*. *pseudomallei* in the environment using sub-genomic methods, such as pulsed-field gel electrophoresis (PFGE) [12] and multi-locus sequence typing (MLST) [13,14]. Although one study found 4 distinct sequence types (STs) within a single soil sample in Ubon, Thailand [12], the resulting data did not identify genomic and phylogenetic differences within each ST. Another study surveyed genotypic diversity with sub-genomic methods in Northeastern Thailand and found 7 distinct STs across 11 soil samples [13]. A study on MLST types in Australia identified that the diversity of *B*. *pseudomallei* populations increased with sampling area, suggesting localized adaptation [15]. A recent study used whole genome sequencing to identify genomic signatures that differ between clinical and environmental *B*. *pseudomallei* isolates in Northeastern Thailand [16]; however, the focus of this study was the overlap between clinical and environmental isolates and not the within-ST diversity of *B*. *pseudomallei*.

The *B*. *pseudomallei* genome is highly plastic [17], owing largely to the acquisition of genomic islands (GIs) [18], which are often acquired from other species via horizontal gene transfer [18]. These GIs generally consist of several contiguous genes and account for an average 5.8% of an individual *B*. *pseudomallei* genome [19]. Previous studies have demonstrated high levels of homologous recombination throughout the *B*. *pseudomallei* genome, including within housekeeping genes [20]; research has shown that recombination is the major driver of evolution in *B*. *pseudomallei* [19] and a diverse and adaptable genome is crucial for its environmental adaptation and success within soil [14,21,22]. Understanding the accessory genome of *B*. *pseudomallei* isolates collected from a limited geographic region may provide a better understanding of its ability to adapt to local ecological conditions.

Multiple mechanisms associated with pathogenesis and virulence have been described in *B*. *pseudomallei*. One of the most characterized virulence factors is the cluster 1 type VI secretion system (T6SS) [23], although the type III secretion system cluster 3 also has been associated with virulence [9]. Recent studies associated a filamentous hemagglutinin (FhaB3) with septic shock and mortality in Australia [24] and the oxidoreductase membrane protein DsbB with virulence based on a knock-out study and subsequent animal challenge [25]. Although the characterization of these factors has improved our understanding of *B*. *pseudomallei* pathogenesis, most mechanisms remain unidentified and virulence in this species remains poorly understood [26,27]. These types of analyses are further confounded by host factors associated with melioidosis [28], suggesting that an interplay of pathogen and host factors are associated with disease severity and patient outcome [8].

The purpose of this study was to examine the evolutionary dynamics of environmental *B*. *pseudomallei* and investigate differential virulence from a single soil sample from Thailand. We sequenced 169 *B*. *pseudomallei* genomes from the soil sample out of a set of 288 isolates. A total of 30 isolates were selected for a murine infection study and comparative genomics and machine learning were used to identify differences between differentially-virulent groups. The results have implications for environmental sampling strategies for *B*. *pseudomallei* that are focused on understanding the virulence potential of clinical and environmental isolates. The characterization of genomic diversity and virulence of sympatric isolates will aid in the understanding of the evolution, ecology, and disease potential of this important human pathogen.

## Methods

### Ethics statement

Mouse challenge experiments were approved by the Animal Care and Use Committee of Colorado State University (CSU), protocol number 17-7497A. The experiments were performed under Select Agent and ABSL-3 containment practices at CSU and in strict accordance with the recommendations in the Guide for the Care and Use of Laboratory Animals of the National Institutes of Health. Every effort was made to minimize animal suffering and pain.

#### *B*. *pseudomallei* isolation

**Fig 1:**
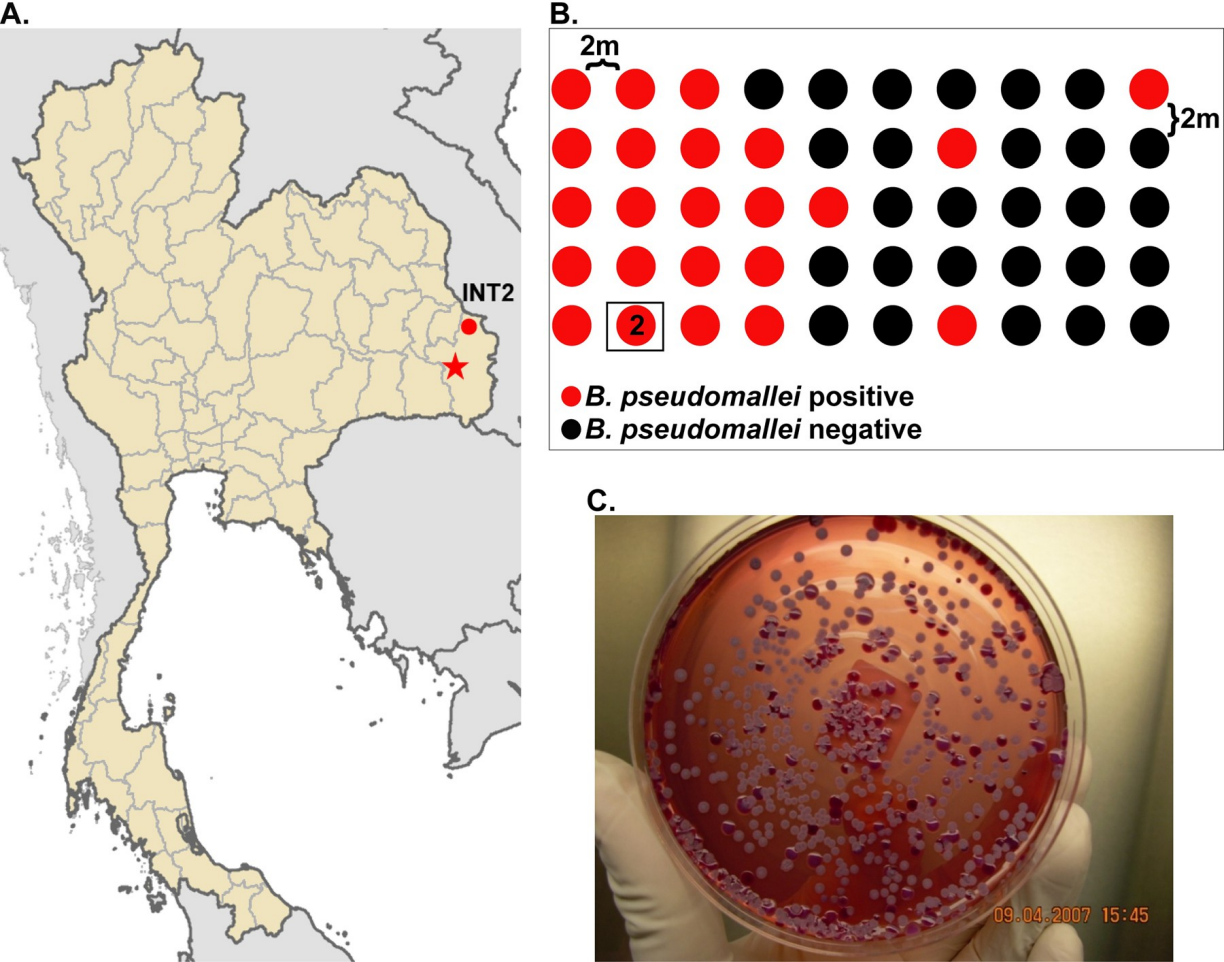
The sampling strategy performed in this study. A) The location in Thailand (circle) where samples were obtained; the city of Ubon Ratchathani within Ubon Ratchathani province is marked by the star. The map of Thailand was created using ArcGIS software by Esri. ArcGIS and ArcMap are the intellectual property of Esri and are used herein under license, all rights reserved, B) the sampling strategy at the intensive sampling site; the location where the INT2 sample was collected is marked and boxed, C) a plate showing the *B*. *pseudomallei* colonies grown out of the single enriched INT2 soil sample; a total of 288 isolates were picked from this plate, and 169 of these isolates were sequenced.

A single site was chosen in the Ubon Rachathani Province (a.k.a. Ubon) of Thailand for sample collection (Fig 1A). The site, a rice field, was sampled in the dry season in a region where *B*. *pseudomallei* is highly endemic. The map of Thailand was created using ArcGIS software by Esri. ArcGIS and ArcMap are the intellectual property of Esri and are used herein under license, all rights reserved. The site was divided into a grid of 50 holes, each separated by 2m (Fig 1B). On 2 April 2007, soil was collected from all 50 holes at a depth of both 10cm and 30cm with a total of 100g of soil collected per sample; we report the results from just one of these 100 samples here as it yielded the largest number of *B*. *pseudomallei* isolates. All soil samples were mixed with 100ml of sterile distilled water in zipped bags and the bags were left to stand at room temperature overnight. A direct plating technique was used to isolate *B*. *pseudomallei* by inoculating 2 different volumes of the soil solution, 10 and 100μl, onto an Ashdown agar plate. The inoculum was spread evenly on the agar plate with an L-shape spreader while the plate was being spun on a plate rotator. The plates were incubated at 42°C for 7 days and were checked daily for growth. A latex agglutination test [29] was used as a presumptive test for *B*. *pseudomallei* identification from ~300 isolates collected from INT2 (Fig 1C). Each suspected *B*. *pseudomallei* colony was picked by a sterile loop and inoculated into each well of a 96-well plate containing 100μl of LB agar. The plate was sealed with adhesive aluminum foil and stored at room temperature before being shipped to Northern Arizona University under a CDC/APHIS permit. Isolates were picked, added to 70% glycerol, and stored at -80°C.

#### DNA extraction and sequencing

Isolates were grown on LB agar at 37°C for 24–48 hours and DNA was extracted from each isolate using the Qiagen DNeasy Blood and Tissue Extraction kit (Qiagen, Hilden, Germany) following the Gram-positive protocol with the addition of 1mg/ml of lysozyme and doubling the volumes. DNA was prepared for multiplexed, paired-end sequencing with a 500 base pair insert using Standard PCR Library Amplification (KAPA Biosystems, Woburn, MA). Genomes were sequenced on either a 2x100 bp paired-end HiSeq run or a 2x250 bp MiSeq run.

#### Genomic assembly and *in silico* genotyping

Illumina sequence data were assembled with the SPAdes assembler v.3.10.0 [30]. The per contig coverage was determined by mapping reads against contigs with Minimap2 v2.17 [31] and calculating coverage with Samtools v1.9 [32]. Each genome assembly was manually edited to remove contigs that had an anomalously low coverage compared to the rest of the assembly or aligned against known contaminants based on a BLASTN alignment [33] against the GenBank [34] nt database. Assembly statistics and accession numbers are shown in S1 Table. The multi-locus sequence type (MLST) for each isolate was calculated from each genome sequence with stringMLST [35] (S1 Table). Representative genome assemblies were submitted to GenBank (S1 Table).

#### Comparative pan-genomics

All genome assemblies were annotated with Prokka v1.14.6 [36]. The pan- and core-genomes were calculated for each ST with Panaroo v1.2.3 [37]. The resulting pan-genome representative sequences were mapped against genome assemblies with the large-scale BLAST score ratio (LS-BSR) tool v1.2.2 [38] in conjunction with BLAT v36x2 [39]. Variably-conserved genes, based on blast score ratio (BSR) values [40], were mapped against phylogenies from each ST with LS-BSR/BLAT and visualized with the interactive tree of life (iTOL) [41].

#### Single nucleotide polymorphism (SNP) identification and phylogenetics

To understand the placement of INT2 genomes within the global *B*. *pseudomallei* phylogeny, INT2 genome assemblies were combined with a global set of *B*. *pseudomallei* genomes (S2 Table); genomes were downloaded using the ncbi-genome-download tool (https://github.com/kblin/ncbi-genome-download) on December 13, 2019. SNPs were called with nucmer v3.1 [42] in conjunction with NASP v1.0.2 [43] for genome assemblies using *B*. *pseudomallei* K96243 (accession number GCA_000011545.1) [44] as the reference. A phylogeny was inferred on the concatenated SNP alignment with FastTree2 v2.1.10 [45]. For all other phylogenies, raw reads were aligned against *B*. *pseudomallei* K96243 with Minimap2 and SNPs were called using the HaplotypeCaller method in GATK v4.1.2 [46,47]. These functions were wrapped with NASP and positions were filtered if the coverage was <10X or the minimum allele frequency ratio was <0.90. All remaining polymorphic positions were concatenated into a single multi-FASTA file. Phylogenetic trees were inferred on concatenated SNP alignments with a maximum-likelihood algorithm implemented in IQ-TREE v1.6.1 [48] using the integrate model search method [49]. The Retention Index of SNP alignments, which provides information on the extent of homoplasy, was calculated with Phangorn v2.4 [50]. The functional annotation of SNPs was determined with SnpEff v4.3t [51]. Homoplastic SNPs were identified from the FASTA file with HomoplasyFinder [52].

#### Population structure

The NASP SNP matrix including the 169 *B*. *pseudomallei* genomes sequenced in this study was used as input into fastSTRUCTURE v1.0 [53] in order to investigate population structure. Using a variational Bayesian framework, the most likely number of populations as well as the probability that each strain belonged to each population was calculated. Only bi-allelic sites were included in this analysis and no phenotype data were provided for this sample set. The admixture model was applied assuming SNPs were unlinked. Individual ancestry and correlated allele frequencies were simulated between the range of K = 2 to K = 10 populations. Individual marginal likelihood values from each K simulation were compared to infer the most likely number of populations (K with the lowest likelihood) using the chooseK.py script included with fastSTRUCTURE [53].

#### Beast timing analysis

BEAST analysis included all 169 genomes sequenced in this study. Putative recombinant SNPs were identified and removed with Gubbins v2.4.1 [54] prior to running BEAST. A SNP matrix based on *B*. *pseudomallei* chromosome I (BX571965.1) that included 5,252 total SNPs with 3,059 parsimony-informative SNPs was analyzed within a Bayesian framework separate from chromosome II (BX571966.1) data based on previous research [55]. The chromosome II dataset included 3,760 total SNPs with 2,484 informative SNPs used in the Bayesian analysis. Additionally, each ST was run through BEAST separately as well using a previous reported evolutionary rate for chromosome I and chromosome II (9.22E^-7^ and 6.71E^-7^ substitutions per site per year, respectively) [55]. To estimate the most recent common ancestor (TMRCA) for the INT2 isolates, a Bayesian strict clock was applied in BEAST version 1.8.4 [56]. MEGA7 [57] was used to infer the best nucleotide substitution model using the Bayesian information criterion (*BIC*) (GTR for chromosome I data and HKY for the chromosome II dataset). A correction for invariant sites was implemented in all BEAST analyses by specifying a Constant Patterns model. Additionally, a “path and stepping stone” sampling marginal-likelihood estimator was used to determine best-fitting clock (strict and relaxed) and demographic model combination (Constant, Bayesian Skygrid, Bayesian Skyline, Extended Bayesian Skyline Plot, GMRF) from ten clock and model combinations [58]. However, no appreciable difference of the marginal likelihood calculation was observed. We implemented the strict skyline clock and skyline demographic model combination to avoid overparameterization. Four independent chains of 1 billion iterations were run for the strict clock and skyline demographic model combination. The program Tracer v1.6.0 [59] was used to visually confirm convergence.

#### *In vivo* animal challenge

Female BALB/c mice, 6–8 weeks old, were purchased from Charles River laboratories and housed at Colorado State University. To assess virulence of a subset of 30 of the soil isolates collected from INT2, each isolate was grown at 37°C in BHI medium to an OD_600_ of 1, supplemented with glycerol to 10% v/v, and frozen in multiple aliquots at -80°C. A vial of these stocks was thawed and serial dilutions plated on BHI agar plates to determine titer 2 days prior to the animal challenge. On the day of the animal challenge, a new frozen aliquot was thawed and diluted with PBS to obtain approximately 500 CFU per 50μL of the bacterial inoculum. The development of the subcutaneous (SC) model in BALB/c mice found an LD_50_ of 1000 CFU [60]; this guided the use of 0.5x LD_50_ in this study to identify differential virulence. Each mouse (5 mice per strain) was subcutaneously inoculated in the medial right leg with 50μL of the bacterial inoculum; subcutaneous infection in mouse models represents percutaneous human infections, which are common in endemic areas [61]. The same volume was also back-titrated to confirm the given dose. In addition to INT2 isolates, mice were also challenged with control strains, NCTC13178 and NCTC13179 [60], at the same dose; strains were purchased from the National Collection of Type Culture (NCTC) in the UK. Mice were weighed and evaluated for clinical symptoms associated with *B*. *pseudomallei* for 21 days post infection. This monitoring was performed daily for the first 7 days and subsequently every 3–4 days to 21 days. The clinical scores indicated in the study were: 0 = normal, 1 = questionable illness, 2 = mild but definitive illness, 3 = moderate illness, 4 = severe illness, moribund-euthanized, 5 = found dead. The moribund and surviving mice at 21 days post infection were euthanized by a standard CO_2_ overdosing method. To compare the virulence of selected isolates, combined survival curves for each ST were graphed using the program GraphPad Prism version 7.00 for mac (GraphPad Software, La Jolla California, USA).

#### Comparative genomics of animal-challenged isolates

Genomes from virulent (*n* = 8) (killed all mice) and attenuated (*n* = 20) (killed no mice) strains, as determined by the mouse challenge, were processed with LS-BSR using BLAT to align coding region sequences (CDS) predicted by Prokka and clustered with CD-HIT v4.8.1 [62]; intermediate virulence strains (*n* = 2) were not included in this analysis. CDSs were identified that were more conserved in either the virulent or attenuated phenotypes with the compare_BSR.py script in the LS-BSR repository based on BSR values; the differential conservation of genes was verified thorough short read mapping approaches where the breadth of coverage was calculated with Samtools. For comparison, the pan-genome was also determined with Panaroo using Prokka-annotated genomes.

#### Genome-wide association analysis (GWAS) with pyseer

To identify genomic regions associated with differential virulence, a Kmer-based GWAS analysis was conducted across animal-challenged genomes. Briefly, assembled genomes were fragmented into 54 base pair Kmers using bbmap (https://sourceforge.net/projects/bbmap/) and Kmers with a frequency <4X coverage in any one genome were converted to “0”; only Kmers that were present in at least one genome were processed further. Kmers were then analyzed with pyseer [63] using default settings with phenotypic data represented by “1” for virulent and “0” for attenuated isolates. For the investigation of genes that contain associated Kmers, regions were extracted from BLASTN alignments, aligned with MUSCLE v3.8.31 [64], and visualized with Jalview [65].

#### Machine learning approaches for feature identification

In order to identify genes associated with virulence, a machine learning algorithm (Boruta) [66] was applied to a binary LS-BSR matrix; a BSR value ≥0.8 for a CDS was coded as present (“1”), whereas a score <0.8 for a CDS was coded as absent (“0”). Of the 7 parameters the Boruta_py module (https://github.com/scikit-learn-contrib/boruta_py) requires, all were kept at default except the estimator object, which is a supervised learning model in which feature importance can be calculated. The percentage strength of Boruta’s shadow features compared to the real features was also calculated, which was set to 99 instead of the default 100 to identify more potential associations. A random forest classifier from the sci-kit learn [67] module was tuned over 250 iterations of stratified 5-fold cross-validation with gridsearchCV and chosen for the estimator object. Finally, the Boruta algorithm was run for 125 iterations over the random seed parameter of the Boruta_py module on the tuned random forest model. Regions of interest were identified by only investigating features that were selected in 100% of the Boruta iterations.

#### Screen of previously-characterized virulence genes

The peptide sequences of 551 genes previously associated with virulence in *B*. *pseudomallei* [68] were screened against the 30 animal-challenged genomes with TBLASTN [69] in conjunction with LS-BSR.

## Results

The diversity of *B*. *pseudomallei* within a single soil sample (intensive site 2, INT2) from the Ubon Ratchathani Province of Thailand was investigated by whole genome sequencing and comparative genomics. An analysis of 169 genomes demonstrated that seven different sequence types (STs) were observed, although three of the STs were single-locus variants of STs observed in INT2 genomes (S1 Table). The differential virulence of a subset of 30 of these isolates was investigated in a murine melioidosis model.

### Global phylogenetics

**Fig 2:**
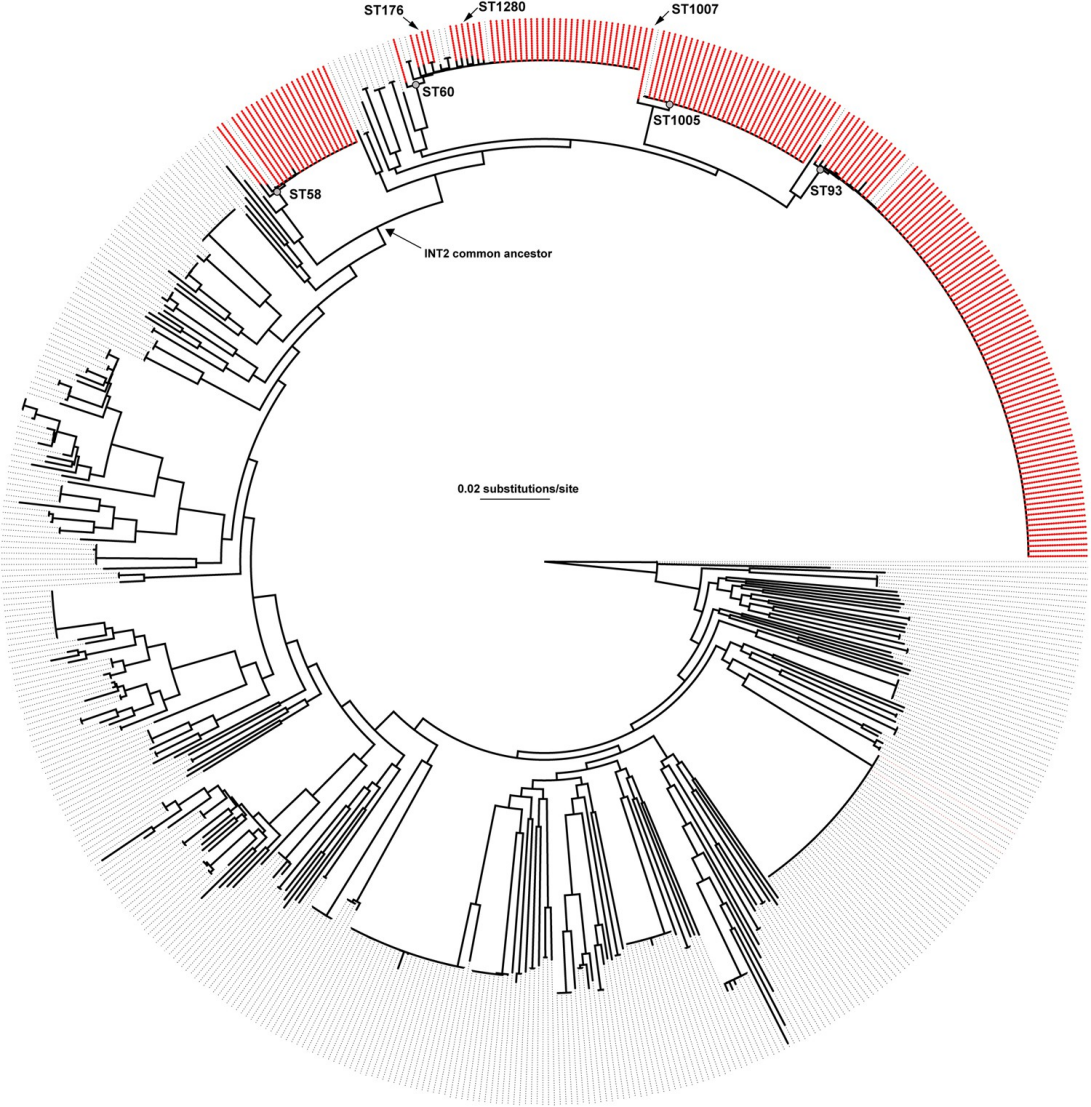
An approximate maximum-likelihood phylogenetic tree inferred with FastTree2 [45] from a concatenation of 90,518 core genome SNPs called from 169 genomes sequenced in this study (red text) as well as 1,576 reference *B*. *pseudomallei* genomes from GenBank (S2 Table). Genomes sequenced in this study are shown in red. The tree was rooted with MSHR668 based on its basal position in other studies [71].

To understand the global diversity of sequenced genomes, the 169 INT2 genomes were compared to 1,576 globally-diverse *B*. *pseudomallei* genomes available in GenBank (S2 Table). The core genome phylogeny demonstrated that genomes from this study shared a recent common ancestor (Fig 2), suggesting a common geographic origin. The retention index (RI) for the concatenated SNP alignment and phylogeny was calculated at 0.8378, indicating homoplasy throughout the *B*. *pseudomallei* core genome, which was expected based on previous studies [70].

### Population genomics of INT2 genomes

**Fig 3:**
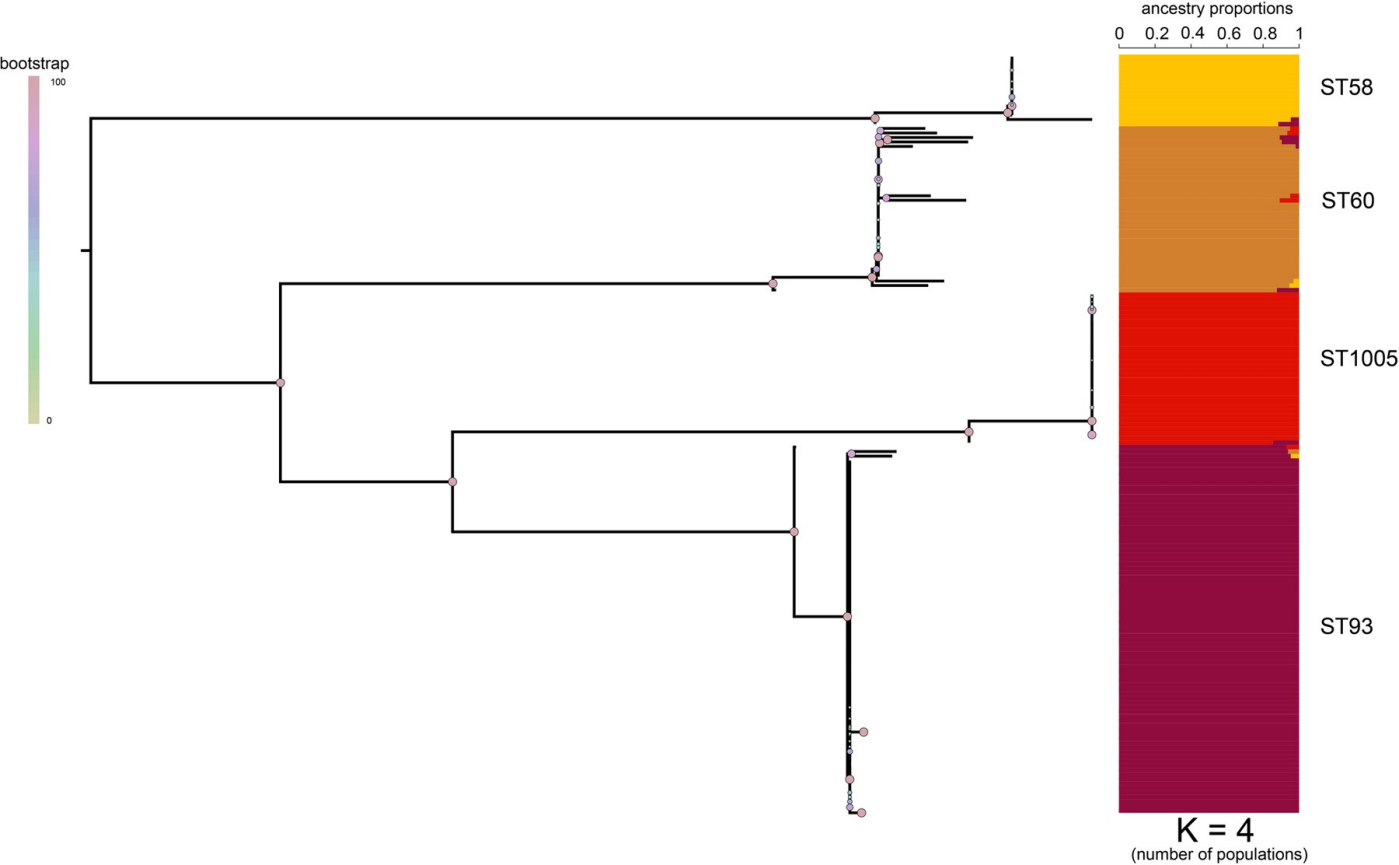
The population structure of INT2 genomes, as revealed by fastSTRUCTURE [53]. The maximum likelihood tree was inferred with IQ-TREE [48] on 22,080 concatenated SNPs using the TVM+F+ASC+R2 substitution model [49]. Four clear populations are present, corresponding to ST58, ST60, ST1005, and ST93, with limited admixing between STs. Each color represents a population and each individual genome is displayed as a horizontal line subdivided into color blocks whose lengths represent the admixture proportions from K = 4 populations.

To better understand their genetic backgrounds, we determined the population structure of the 169 *B*. *pseudomallei* genomes from INT2 using Bayesian methods implemented in fastSTRUCTURE. By comparing individual marginal likelihood values to each individual K value (signifying number of populations), the most reliable population distribution was determined. The marginal likelihood values identified K = 4 as the best scenario with four distinct populations largely corresponding to ST58, ST93, ST1005, and ST60 (Fig 3); these populations are highly correlated with phylogenetic groupings (Figs 2 and 3). The ST58 population is composed of a mostly unique clade (solid yellow) with isolates Bp1763 and Bp1752 representing recombination with the ST93 clade. The ST60 population is composed of admixtures between genomes from ST93, ST1005, and ST58 populations. The ST1005 population is represented by a unique genetic clade (solid red) with only one genome (Bp1818; ST1007) displaying an admixture. Furthermore, while ST93 has the largest number of genomes (n = 82), only three isolates displayed an admixture (Bp1740, Bp1805, Bp8880).

A concatenated SNP alignment from 169 INT2 genomes identified 7,960 core genome SNPs, out of 22,080 total SNPs, with a consistency index ≤0.5. The locus tags in K96243 with the highest number of homoplastic SNPs in INT2 genomes include: BPSS1007 (polyketide synthase), BPSS0409 (hypothetical protein), BPSS0306 (polyketide synthase), and BPSL1712 (non-ribosomal antibiotic-related protein synthase); the functions of these and many other regions containing homoplastic SNPS are largely unknown.

### Comparative genomics of representative STs

Sub-trees were generated for the four major STs (ST60, ST58, ST1005, ST93) (S1–S4 Figs, respectively). Although gene content variability within each ST was largely due to convergent gene loss, unique genes were also identified in a subset of strains; unique and lost genes are represented as a heatmap correlated to the phylogeny (S1–S4 Figs). A pan-genomics analysis determined that the core and pan-genome for each ST were largely similar in size (Table 1), suggesting minimal, yet observable gene differences within each ST. When compared to the global set of *B*. *pseudomallei* genomes, unique gene variants were identified within each of the four STs (Table 1); although homologs were observed for these unique regions in other *B*. *pseudomallei* genomes, each ST-specific sequence contained unique insertions/deletions that were not observed in these other genomes and, thus, represent genes that encode distinct proteins.

**Table 1:** Coding region stats for each major INT2 sequence type.

Population		Pan-genome size (#CDSs)	Core-genome size (#CDSs)	#Unique variants	
	#genomes				Unique locus tags
93	82	6185	4885	1	AQ771_RS06540
58	16	5834	5604	1	C1W81_11530
1005	34	5896	5674	2	C1W93_05955,C1W93_24425
60	37	5963	5551	2	C1W75_00010,C1W75_27835

### Bayesian timing analysis

The 169 INT2 *B*. *pseudomallei* genomes representing the four populations identified by fastStructure were included in the Bayesian convergence analysis. The SNP matrix included reference positions present in >90% of reads and had a minimum coverage of 10X that spanned 90.1% of reference chromosome I in K96243 (NC_006350.1) and 75.1% of reference chromosome II (NC_006351.1).

**Fig 4:**
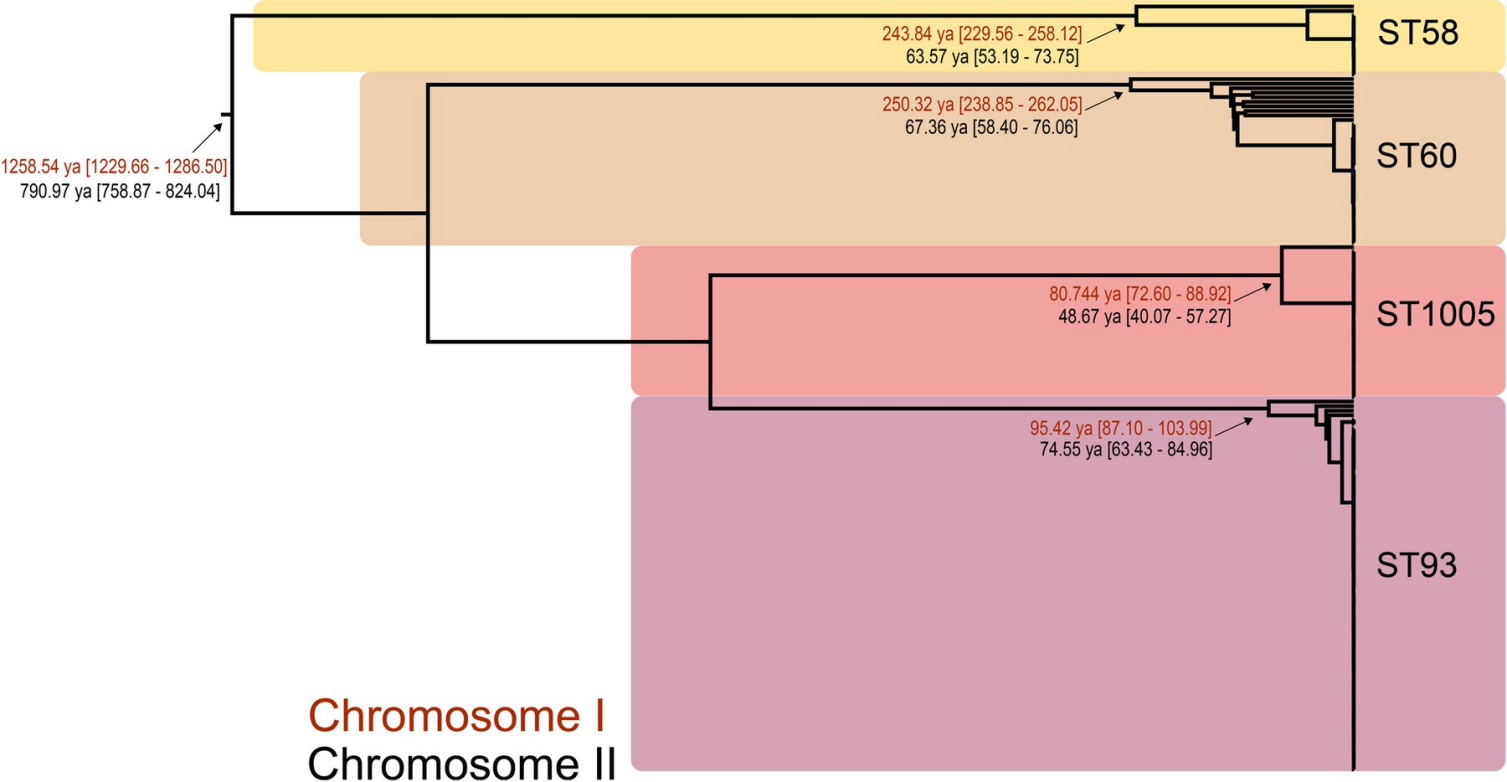
Estimate of times to most recent common ancestor for the 169 INT2 *B*. *pseudomallei* genomes using BEAST [56]. Node dating ranges for both chromosomes (chromosome I in red, chromosome II in black) are shown on the Bayesian phylogeny. STs are indicated for each population.

Based on previous research [55], the estimated mutation rates for chromosome I and chromosome II differ slightly, 9.22E^-7^ vs 6.71E^-7^. As such, the time to most recent common ancestor (TMRCA) was calculated in the BEAST analysis separately for each chromosomal dataset using these previously reported mutation rates. For the 169 INT2 genomes, the mean TMRCA for chromosome I was estimated at 1258.5 years ago (95% highest posterior density (HPD), 1229.7, 1286.5), and the mean TMRCA for chromosome II was estimated at 791.0 years ago (95% HPD, 757.8, 823.1; Fig 4). The TMRCA for the ST1005 clade was estimated at 80.7 years ago (95% HPD, 72.6, 88.9) for chromosome I and 48.7 years ago (95% HPD, 40.1, 57.3) for chromosome II. The TMRCA for ST58 clade was estimated at 243.8 years ago (95% HPD, 229.6, 258.1) for chromosome I and 63.6 years ago (95% HPD, 53.2, 73.8) for chromosome II. The ST93 lineage TMRCA was estimated to be 95.4 years ago (95% HPD, 87.1, 104.0) for chromosome I and 74.5 years ago (95% HPD, 63.4, 85.0) for chromosome II. The ST60 clade TMRCA was estimated to be 250.3 (95% HPD, 238.9, 262.0) for chromosome I and 67.4 years ago (95% HPD, 58.4, 76.1) for chromosome II.

### Differential virulence

**Fig 5:**
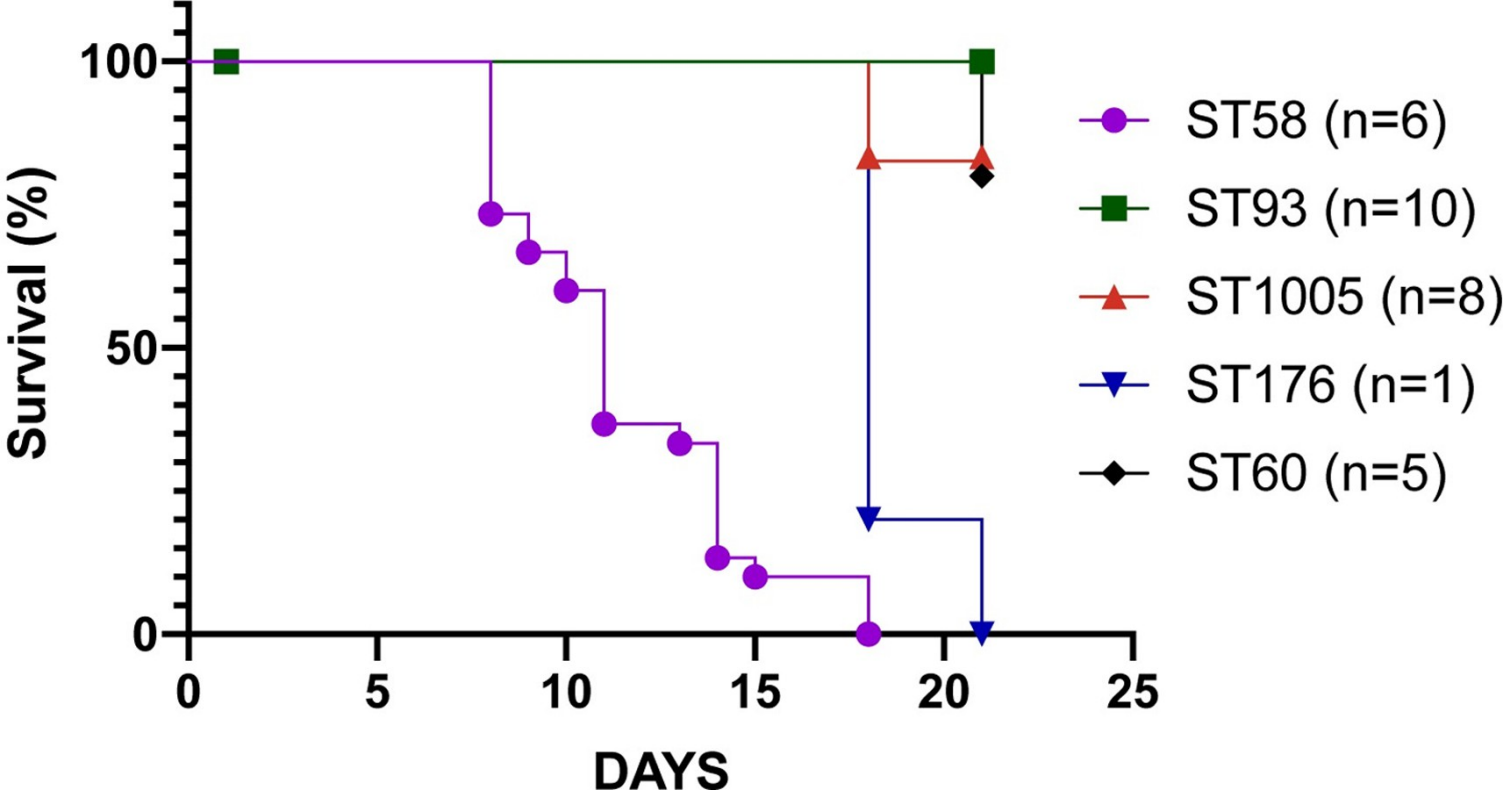
A kill curve based on subcutaneous injection of 500 CFUs in 5 mice for each isolate. The mortality curves were plotted in Prism. For each ST, all mice were pooled, and mortality curves plotted. Raw survival data per isolate is shown in S3 Table.

The virulence of 30 INT2 *B*. *pseudomallei* isolates assigned to five different sequence types was examined in a murine infection model (5 mice per strain); these included isolates from the four main STs (ST58, ST60, ST93, ST1005), as well as isolate Bp8873 from ST176 (S1 Table); ST176 is a single locus variant of ST60. In addition, two controls strains with known variable virulence in this model (NCTC13178, NTCT13179) were processed at the same dosage. The probability of survival was calculated from the pooled challenged mice per tested ST (Fig 5, raw data in S3 Table). A core genome phylogeny of 169 INT2 isolate genomes demonstrates the phylogenetic distribution of the 30 isolates utilized for virulence studies within this larger set (S5 Fig). Isolates were considered virulent if they killed all tested mice, attenuated if they killed no mice, and intermediate if they killed between 1 and 5 mice. None of the tested ST93 isolates (n = 10) killed any mice based on the subcutaneous inoculation pathway. In contrast, all tested ST58 isolates (n = 6) were highly virulent, killing all challenged mice. Interestingly, six ST1005 strains killed no mice, whereas 1 ST1005 strain (Bp8884) killed all mice challenged (Fig 5 and S3 Table). Four ST60 isolates did not kill any challenged mice, whereas one isolate (Bp8881) killed one mouse. The single ST176 isolate (Bp8873) was highly virulent, killing all challenged mice before the end of the 21-day experiment. The virulent control strain (NTCT13178) killed all mice at 500 CFU by day 5 and the less virulent control strain killed no mice over the 21-day experiment.

### Genome wide association studies

To identify genotypic differences that may explain the virulent phenotype, the program pyseer was run on a set of all 54-mers from Illumina reads from each of the 30 INT2 isolates used for virulence studies. Pyseer identified 31 virulence-associated 54-mers, all of which mapped to a single annotated gene, *mgtA* (C1W34_19470). However, all of these Kmers had “bad chisq” values signifying possible spurious associations; a “bad chisq” value is reported by pyseer and no additional information is available. These 31 associated Kmers were conserved in 7 of 8 virulent genomes and 8 of 20 attenuated genomes. However, a manual inspection of an alignment of this gene demonstrated that SNPs were highly correlated with population structure, with the exception of a single SNP found in all ST58 and ST60 genomes (including ST176), most likely due to homologous recombination.

In addition to pyseer, we also used a machine learning (ML) Boruta model to identify CDSs potentially associated with virulence. ML has been previously used to accurately predict phenotype from genotype [72,73]. Although ML methods used in this study have not been previously used for bacterial GWAS, the Boruta method has been established by statisticians and human GWAS researchers as an efficient and specific feature selection algorithm [66,74]. By iterating over the random seed of Boruta, we were able to identify 45 associated CDSs that were consistently selected as associated with virulence, although only 11 of them (S6 Fig) were more conserved in virulent genomes. Some of these regions demonstrate a population structure effect, whereas others represent a polyphyletic distribution, most likely due to recombination.

### Pan-genomics of animal-challenged genomes

A pan-genomics analysis of the 30 INT2 isolates utilized in the virulence study demonstrated a core-genome size of 5,369 CDSs and a pan-genome size of 6,338 CDSs. To identify potential differences in the pan-genome between virulent and attenuated strains, an LS-BSR analysis was performed, which failed to identify any genomic regions that fully differentiated between these two phenotypes. However, at a BSR threshold of 0.75, there were two CDSs (C1W87_17265; hypothetical protein, AQ770_28965; non-ribosomal peptide synthetase) that were highly conserved in the genomes of 7 of 8 virulent isolates and less conserved in the genomes of all 20 attenuated isolates (S6 Fig); these same two regions were also identified by the machine learning method. Of the seven virulent isolates possessing these regions, six were assigned to ST58 and one to ST176 (Bp8873). ST176 is a single locus variant of ST60 and none of the ST60 isolates exhibited the virulent phenotype. The polyphyletic distribution of these 2 genes (S6 Fig) suggests that these regions were a product of recombination and could be the subject of additional investigation.

For ST1005, six of the isolates were attenuated, one demonstrated intermediate virulence, and one of the isolates was virulent. A comparative analysis failed to identify CDSs that fully differentiated between phenotypes within this ST. A Kmer analysis identified 31 Kmers that were associated with a single locus (C1W93_20275) that was enriched in the genome from the virulent isolate; this was the same locus that was identified by pyseer on the complete set of animal-challenged genomes.

A screen of previously characterized virulence factors across the genomes of the 30 INT2 isolates included in the virulence study identified that Hcp1, which has previously been linked with virulence in *B*. *pseudomallei* [23], was associated with the virulent phenotype; this region was also identified with the ML method. All 8 of the virulent strains had the same version of Hcp1 found in K96243 (BPSS1498). However, 14 of 20 attenuated isolates, plus one intermediate virulence isolate (Bp8881), contain a variant of Hcp1 (C1W89_RS19320) with significant amino acid differences to BPSS1498 (S7 Fig). This demonstrates that this Hcp1 homolog has a different peptide composition and may have a different protein activity associated with attenuated virulence. A screen of the nucleotide sequences of Hcp1 against all ST93 genomes in the GenBank assembly database demonstrated that both alleles are present in INT2 ST93 genomes (S8 Fig), demonstrating recombination of this region; INT2 genome Bp1782, in particular, has an annotated Hcp1 that is highly divergent at both the nucleotide and amino acid level (identical to another annotated Hpc1 gene (BBU_3899)), suggesting that it may have altered function. Six attenuated strains from other STs (Bp8894, Bp8892, Bp8871, Bp8890, Bp8886, Bp1927) had the K96243 version of Hcp1, suggesting that a different mechanism of attenuation exists in those strains. Interestingly, Panaroo grouped both Hcp1 variants (BPSS1498, C1W89_RS19320) together into a single gene and the binary presence/absence results demonstrate that all animal-challenged genomes contain the K96243 Hcp1 allele; using Panaroo alone on this dataset would not have identified the ST93 Hcp1 differences.

For the remaining 6 attenuated ST1005 isolates with the K96243 Hcp1 allele (Bp8892, Bp8886, Bp8890, Bp8894, Bp8871, Bp1927), a comparison was performed with all virulent INT2 isolate genomes (n = 8) using a set of previously characterized virulence genes in *B*. *pseudomallei*. The results demonstrate that a cluster of genes (BPSS0417-BPSS0428) associated with virulence was conserved in 7 of 8 virulent isolates from the other STs (BSR value ≥0.8) and was missing from all ST1005 isolates (6 attenuated and 1 virulent isolates); these genes are all part of a polysaccharide biosynthetic operon that has been demonstrated to react strongly with patient sera [75].

## Discussion

In this study, we explored the genomic diversity and differential virulence of *B*. *pseudomallei* within a single soil sample collected from a rice paddy in the Ubon Ratchathani Province of Thailand. MLST analysis identified 7 unique sequence types (STs) within a single soil sample, highlighting genotypic diversity and prompting the investigation into whole genome comparisons and *in vivo* animal challenge.

Comparative genomics identified variably conserved coding region sequence (CDS) differences between STs as well as within STs. Genomes from a single ST were not monomorphic, demonstrating within-ST diversity from a single soil sample. The within-ST diversity in these isolates was only apparent by sequencing multiple bacterial colonies from the same isolation plate. If only a single colony was picked from this soil sample during propagation, the comprehensive sample diversity described here would have been missed. If a plate sweep had been sequenced, a ST mixture would have been recovered and would have complicated all downstream population analyses. As demonstrated by this study, an analysis of individual colonies was the best option to identify and understand the sample diversity and should be considered for comprehensive environmental sampling of *B*. *pseudomallei*.

An investigation into population structure identified 4 distinct populations (Fig 3), with demonstrated mixing between a small number of genomes. Horizontal gene transfer and recombination have been demonstrated to help drive the diversification in *B*. *pseudomallei* [19], and the observation of recombination in genomes from a single sample is not surprising given their proximity and related genomic background. The CDSs that demonstrated the highest levels of homoplasy were associated with non-ribosomal protein synthetases; the function of many of these regions is not known but suggests potential mechanisms of environmental adaptation. Other recombination events were observed in virulence-associated genes (S6 Fig), including in Hcp1, a gene associated with secretion of type six effectors and disease in mammals. This result suggests that recombination could drive virulence in environmental *B*. *pseudomallei* strains.

The subcutaneous (SC) BALB/c model was used to identify variable virulence as it simulates percutaneous infection [7], has published LD_50_ values [60], and has been used in other virulence studies [76,77]. We did not perform histology in this study and it is unclear if surviving mice were colonized by *B*. *pseudomallei*. The results do demonstrate that different strains kill mice at a similar dosage, which may correlate to variable disease presentation in human infections. Variable virulence was observed among isolates from INT2 and was largely segregated by ST. For example, ST93 was found to be highly attenuated in the mouse model based on the dosage and route of exposure. A screen of public genomes in GenBank identified 8 ST93 genomes in addition to those sequenced in this study; 6 of these additional ST93 genomes were isolated from the environment whereas the other 2 were reported to be isolated from humans, although details on isolation source were missing. In general, the results of this study demonstrate that ST alone is insufficient to predict virulence in *B*. *pseudomallei*. A recent study identified genomic differences between clinical and environmental isolates [16], suggesting that some environmental isolates may not contain genomic elements required for virulence in humans.

The animal study prompted analysis into genes associated with the virulent phenotype. A screen of genes previously associated with virulence identified that all attenuated ST93 genomes have an alternative allele for Hcp1, an important virulence factor in *B*. *pseudomallei*. Of external ST93 genome assemblies, 3 have the K96243 version of Hcp1, while 5 have the alternate allele; one of the external genomes with the alternate Hcp1 allele was associated with humans (SAMN04208633), although details of the isolation source are not available in the public record. Previous results demonstrated that even single amino acid differences can result in different phenotypes against a similar genomic background [78]. Although most ST93 genomes sequenced in this study (80/82) have the alternative Hcp1 allele, two genomes have an identical allele to Hcp1 in *B*. *pseudomallei* K96243 (S8 Fig); the presence of both alleles within a single soil sample is most likely explained by homologous recombination. Testing these naturally occurring, related strains in a murine melioidosis model will demonstrate if this one allele difference is responsible for the differential virulence observed in this sequence type; if virulence is observed in the wild strain, allele replacement will confirm that Hcp1 confers the virulent phenotype. For other genomes associated with virulence, machine learning methods identified additional regions that were not strictly associated with population structure, suggesting that the apparent recombination may be associated with the observed virulence phenotype in the murine model, but additional experimentation is required to validate these results and benchmark the Boruta ML method. The lack of a single region that explains the virulence phenotype suggests that either multiple independent mechanisms are associated with virulence or that complex interactions between mechanisms result in the virulent phenotype.

Our findings indicate that environmental exposure to *B*. *pseudomallei*, especially inoculation via contact with contaminated soil and water, but possibly also via inhalation and ingestion, likely means exposure to a mixed community of *B*. *pseudomallei* strains. Attenuated strains are likely to be quickly cleared by the host immune system, although the virulent strains may cause disease in sensitive patients. A recent longitudinal study of melioidosis in a single patient demonstrated the selection for specific genotypes with significantly reduced virulence in chronic infections [78], suggesting that either the immune system can select for strains from the environment that cause attenuated, but chronic infection, or that strains that evade the immune system persist in the human host.

In addition to being a significant public health threat, *B*. *pseudomallei* is also a biothreat agent and our results have implications for studies of the pathogen in this context. The diversity of INT2 isolate genomes, measured by total pairwise SNPs as well as pan-genome size, is greater than the known, global diversity of other biothreat pathogens, including *Bacillus anthracis* [79], *Yersinia pestis* [80], and *Francisella tularensis* [81]. This diversity complicates sample matching [82], suggesting that matching disease to a specific strain using shared mutations may need to be supplemented by overlap in the accessory genome. A recent study identified unique genomic islands in isolates from Puerto Rico [10], suggesting that whole genome comparisons are needed for robust sample matching.

This study presents a focused survey into the evolution, diversity, and virulence of *B*. *pseudomallei* isolates from a single soil sample. Based on the projected distribution of *B*. *pseudomallei* throughout the world [83], there likely exists an underexplored diversity of strains that have variable risk to humans. This study provides a framework for studying this diversity and identifies genomic targets that may focus additional studies to better understand the virulence of *B*. *pseudomallei*.

## Supporting information

S1 Fig A maximum-likelihood phylogeny of ST93 genomes inferred from a concatenation of 15,249 SNPs with IQ-TREE [48] using the TVM+F+ASC+R3 substitution model [49]. The phylogeny was annotated with coding regions that show variable distribution, based on an analysis with LS-BSR [38]. The phylogeny and heatmap were visualized with the interactive tree of life [41] and rooted with *B*. *pseudomallei* K96243 [44] as it represents an outgroup genome from Thailand.
(TIF)

S2 Fig A maximum-likelihood phylogeny of ST58 genomes inferred from a concatenation of 27,500 SNPs with IQ-TREE [48] using the GTR+F+ASC+R2 substitution model [49]. The phylogeny was annotated with coding regions that show variable distribution, based on an analysis with LS-BSR [38]. The phylogeny and heatmap were visualized with the interactive tree of life [41] and rooted with *B*. *pseudomallei* K96243 [44] as it represents an outgroup genome from Thailand.
(TIF)

S3 Fig A maximum-likelihood phylogeny of ST60 genomes inferred from a concatenation of 23,735 SNPs with IQ-TREE [48] using the TVM+F+ASC+R4 substitution model [49]. The phylogeny was annotated with coding regions that show variable distribution, based on an analysis with LS-BSR [38]. The phylogeny and heatmap were visualized with the interactive tree of life [41] and rooted with *B*. *pseudomallei* K96243 [44] as it represents an outgroup genome from Thailand.
(TIF)

S4 Fig A maximum-likelihood phylogeny of ST1005 genomes inferred from a concatenation of 20,923 SNPs with IQ-TREE [48]. The phylogeny was annotated with coding regions that show variable distribution, based on an analysis with LS-BSR [38] using the TVMe+ASC+R3 substitution model [49]. The phylogeny and heatmap were visualized with the interactive tree of life [41] and rooted with *B*. *pseudomallei* K96243 [44] as it represents an outgroup genome from Thailand.
(TIF)

S5 Fig A maximum-likelihood phylogeny of all sequenced INT2 genomes (n = 169) inferred from a concatenation of 22,080 SNPs with IQ-TREE [48] using the TVM+F+ASC+R2 substitution model [49]. Animal-passed genomes are colored by attenuated, intermediate, or virulent phenotypes. The phylogeny is rooted by *B*. *pseudomallei* K96243 [44] as it represents an outgroup genome from Thailand.
(TIF)

S6 Fig A maximum-likelihood phylogeny of genomes from animal-challenged INT2 isolates inferred from a concatenation of 32,821 SNPs with IQ-TREE [48] using the TVM+F+ASC substitution model [49]. Genomes are colored by the virulence outcome in an animal challenge model. Each genome was screened with LS-BSR [38] using genes identified through machine learning methods. The phylogeny and heatmap were visualized with the interactive tree of life [41] and rooted with *B*. *pseudomallei* K96243 [44] as it represents an outgroup genome from Thailand.
(TIF)

S7 Fig An alignment of protein sequences from Hcp1 (BPSS1498) and a novel allele variant seen in ST93 genomes (C1W89_RS19320). Blue boxes surround amino acid differences in the alignment, which was visualized with Jalview [65].
(TIF)

S8 Fig A maximum-likelihood phylogeny of ST93 genomes sequenced in this study as well as GenBank external genomes inferred from a concatenation of 37,746 SNPs with IQ-TREE [48] using the TVM+F+ASC substitution model [49]. The phylogeny was rooted with *B*. *pseudomallei* K96243 [44]. Each genome was screened with LS-BSR [38] using two Hcp1 variants. The phylogeny and heatmap were visualized with the interactive tree of life [41] and rooted with *B*. *pseudomallei* K96243 [44] as it represents an outgroup genome from Thailand.
(TIF)

S1 Table Accession information for genomes sequenced in this study. (XLSX)

S2 Table Accession information for a global set of *B. pseudomallei* genomes. (XLSX)

S3 Table Number of mice remaining after each day of the mouse challenge experiment. (XLSX)

## References

[1] Peacock SJ. Melioidosis. Current opinion in infectious diseases. 2006;19(5):421–8. doi: 10.1097/01.qco.0000244046.31135.b3 . 16940864

[2] Currie BJ, Ward L, Cheng AC. The epidemiology and clinical spectrum of melioidosis: 540 cases from the 20 year Darwin prospective study. PLoS neglected tropical diseases. 2010;4(11):e900. doi: 10.1371/journal.pntd.0000900 ; PubMed Central PMCID: PMC2994918. 21152057 PMC2994918

[3] Douglas MW, Lum G, Roy J, Fisher DA, Anstey NM, Currie BJ. Epidemiology of community-acquired and nosocomial bloodstream infections in tropical Australia: a 12-month prospective study. Trop Med Int Health. 2004;9(7):795–804. doi: 10.1111/j.1365-3156.2004.01269.x . 15228489

[4] Currie BJ. Melioidosis: an important cause of pneumonia in residents of and travellers returned from endemic regions. Eur Respir J. 2003;22(3):542–50. Epub 2003/10/01. doi: 10.1183/09031936.03.00006203 . 14516149

[5] Dance D. Treatment and prophylaxis of melioidosis. Int J Antimicrob Agents. 2014;43(4):310–8. Epub 2014/03/13. doi: 10.1016/j.ijantimicag.2014.01.005 ; PubMed Central PMCID: PMC4236584. 24613038 PMC4236584

[6] White NJ. Melioidosis. Lancet. 2003;361(9370):1715–22. Epub 2003/05/28. doi: 10.1016/s0140-6736(03)13374-0 . 12767750

[7] Currie BJ, Fisher DA, Howard DM, Burrow JN, Selvanayagam S, Snelling PL, et al. The epidemiology of melioidosis in Australia and Papua New Guinea. Acta Trop. 2000;74(2–3):121–7. Epub 2000/02/16. doi: 10.1016/s0001-706x(99)00060-1 . 10674639

[8] Cheng AC, Currie BJ. Melioidosis: epidemiology, pathophysiology, and management. Clinical microbiology reviews. 2005;18(2):383–416. Epub 2005/04/16. doi: 10.1128/CMR.18.2.383-416.2005 ; PubMed Central PMCID: PMC1082802. 15831829 PMC1082802

[9] Wiersinga WJ, van der Poll T, White NJ, Day NP, Peacock SJ. Melioidosis: insights into the pathogenicity of Burkholderia pseudomallei. Nature reviews Microbiology. 2006;4(4):272–82. doi: 10.1038/nrmicro1385 . 16541135

[10] Hall CM, Jaramillo S, Jimenez R, Stone NE, Centner H, Busch JD, et al. Burkholderia pseudomallei, the causative agent of melioidosis, is rare but ecologically established and widely dispersed in the environment in Puerto Rico. PLoS neglected tropical diseases. 2019;13(9):e0007727. Epub 2019/09/06. doi: 10.1371/journal.pntd.0007727 ; PubMed Central PMCID: PMC6748447. 31487287 PMC6748447

[11] Stone NE, Hall CM, Browne AS, Sahl JW, Hutton SM, Santana-Propper E, et al. Burkholderia pseudomallei in Soil, US Virgin Islands, 2019. Emerg Infect Dis. 2020;26(11):2773–5. Epub 2020/10/21. doi: 10.3201/eid2611.191577 . 33079041 PMC7588534

[12] Chantratita N, Wuthiekanun V, Limmathurotsakul D, Vesaratchavest M, Thanwisai A, Amornchai P, et al. Genetic diversity and microevolution of Burkholderia pseudomallei in the environment. PLoS neglected tropical diseases. 2008;2(2):e182. Epub 2008/02/27. doi: 10.1371/journal.pntd.0000182 ; PubMed Central PMCID: PMC2254201. 18299706 PMC2254201

[13] Wuthiekanun V, Limmathurotsakul D, Chantratita N, Feil EJ, Day NP, Peacock SJ. Burkholderia Pseudomallei is genetically diverse in agricultural land in Northeast Thailand. PLoS neglected tropical diseases. 2009;3(8):e496. Epub 2009/08/05. doi: 10.1371/journal.pntd.0000496 ; PubMed Central PMCID: PMC2713400. 19652701 PMC2713400

[14] U’Ren J M, Hornstra H, Pearson T, Schupp JM, Leadem B, Georgia S, et al. Fine-scale genetic diversity among Burkholderia pseudomallei soil isolates in northeast Thailand. Appl Environ Microbiol. 2007;73(20):6678–81. Epub 2007/08/28. doi: 10.1128/AEM.00986-07 ; PubMed Central PMCID: PMC2075045. 17720819 PMC2075045

[15] McRobb E, Kaestli M, Price EP, Sarovich DS, Mayo M, Warner J, et al. Distribution of Burkholderia pseudomallei in northern Australia, a land of diversity. Appl Environ Microbiol. 2014;80(11):3463–8. Epub 2014/03/25. doi: 10.1128/AEM.00128-14 ; PubMed Central PMCID: PMC4018869. 24657869 PMC4018869

[16] Chewapreecha C, Mather AE, Harris SR, Hunt M, Holden MTG, Chaichana C, et al. Genetic variation associated with infection and the environment in the accidental pathogen Burkholderia pseudomallei. Commun Biol. 2019;2:428. Epub 2019/12/05. doi: 10.1038/s42003-019-0678-x ; PubMed Central PMCID: PMC6874650. 31799430 PMC6874650

[17] Moule MG, Hemsley CM, Seet Q, Guerra-Assuncao JA, Lim J, Sarkar-Tyson M, et al. Genome-wide saturation mutagenesis of Burkholderia pseudomallei K96243 predicts essential genes and novel targets for antimicrobial development. mBio. 2014;5(1):e00926–13. doi: 10.1128/mBio.00926-13 ; PubMed Central PMCID: PMC3950516. 24520057 PMC3950516

[18] Tuanyok A, Leadem BR, Auerbach RK, Beckstrom-Sternberg SM, Beckstrom-Sternberg JS, Mayo M, et al. Genomic islands from five strains of Burkholderia pseudomallei. BMC genomics. 2008;9:566. doi: 10.1186/1471-2164-9-566 ; PubMed Central PMCID: PMC2612704. 19038032 PMC2612704

[19] Spring-Pearson SM, Stone JK, Doyle A, Allender CJ, Okinaka RT, Mayo M, et al. Pangenome Analysis of Burkholderia pseudomallei: Genome Evolution Preserves Gene Order despite High Recombination Rates. PLoS One. 2015;10(10):e0140274. Epub 2015/10/21. doi: 10.1371/journal.pone.0140274 ; PubMed Central PMCID: PMC4613141. 26484663 PMC4613141

[20] Pearson T, Giffard P, Beckstrom-Sternberg S, Auerbach R, Hornstra H, Tuanyok A, et al. Phylogeographic reconstruction of a bacterial species with high levels of lateral gene transfer. BMC Biol. 2009;7:78. Epub 2009/11/20. doi: 10.1186/1741-7007-7-78 ; PubMed Central PMCID: PMC2784454. 19922616 PMC2784454

[21] Sim SH, Yu Y, Lin CH, Karuturi RK, Wuthiekanun V, Tuanyok A, et al. The core and accessory genomes of Burkholderia pseudomallei: implications for human melioidosis. PLoS Pathog. 2008;4(10):e1000178. Epub 2008/10/18. doi: 10.1371/journal.ppat.1000178 ; PubMed Central PMCID: PMC2564834. 18927621 PMC2564834

[22] Tumapa S, Holden MT, Vesaratchavest M, Wuthiekanun V, Limmathurotsakul D, Chierakul W, et al. Burkholderia pseudomallei genome plasticity associated with genomic island variation. BMC genomics. 2008;9:190. Epub 2008/04/29. doi: 10.1186/1471-2164-9-190 ; PubMed Central PMCID: PMC2386483. 18439288 PMC2386483

[23] Burtnick MN, Brett PJ, Harding SV, Ngugi SA, Ribot WJ, Chantratita N, et al. The cluster 1 type VI secretion system is a major virulence determinant in Burkholderia pseudomallei. Infection and immunity. 2011;79(4):1512–25. Epub 2011/02/09. doi: 10.1128/IAI.01218-10 ; PubMed Central PMCID: PMC3067527. 21300775 PMC3067527

[24] Sarovich DS, Price EP, Webb JR, Ward LM, Voutsinos MY, Tuanyok A, et al. Variable virulence factors in Burkholderia pseudomallei (melioidosis) associated with human disease. PLoS One. 2014;9(3):e91682. Epub 2014/03/13. doi: 10.1371/journal.pone.0091682 ; PubMed Central PMCID: PMC3950250. 24618705 PMC3950250

[25] McMahon RM, Ireland PM, Sarovich DS, Petit G, Jenkins CH, Sarkar-Tyson M, et al. Virulence of the Melioidosis Pathogen Burkholderia pseudomallei Requires the Oxidoreductase Membrane Protein DsbB. Infection and immunity. 2018;86(5). Epub 2018/02/15. doi: 10.1128/IAI.00938-17 ; PubMed Central PMCID: PMC5913862. 29440370 PMC5913862

[26] Memisevic V, Zavaljevski N, Pieper R, Rajagopala SV, Kwon K, Townsend K, et al. Novel Burkholderia mallei virulence factors linked to specific host-pathogen protein interactions. Mol Cell Proteomics. 2013;12(11):3036–51. Epub 2013/06/27. doi: 10.1074/mcp.M113.029041 ; PubMed Central PMCID: PMC3820922. 23800426 PMC3820922

[27] Singh AP, Lai SC, Nandi T, Chua HH, Ooi WF, Ong C, et al. Evolutionary analysis of Burkholderia pseudomallei identifies putative novel virulence genes, including a microbial regulator of host cell autophagy. J Bacteriol. 2013;195(24):5487–98. Epub 2013/10/08. doi: 10.1128/JB.00718-13 ; PubMed Central PMCID: PMC3889600. 24097950 PMC3889600

[28] Suputtamongkol Y, Chaowagul W, Chetchotisakd P, Lertpatanasuwun N, Intaranongpai S, Ruchutrakool T, et al. Risk factors for melioidosis and bacteremic melioidosis. Clin Infect Dis. 1999;29(2):408–13. Epub 1999/09/07. doi: 10.1086/520223 . 10476750

[29] Amornchai P, Chierakul W, Wuthiekanun V, Mahakhunkijcharoen Y, Phetsouvanh R, Currie BJ, et al. Accuracy of Burkholderia pseudomallei identification using the API 20NE system and a latex agglutination test. J Clin Microbiol. 2007;45(11):3774–6. Epub 2007/09/07. doi: 10.1128/JCM.00935-07 ; PubMed Central PMCID: PMC2168515. 17804660 PMC2168515

[30] Bankevich A, Nurk S, Antipov D, Gurevich AA, Dvorkin M, Kulikov AS, et al. SPAdes: a new genome assembly algorithm and its applications to single-cell sequencing. Journal of computational biology: a journal of computational molecular cell biology. 2012;19(5):455–77. Epub 2012/04/18. doi: 10.1089/cmb.2012.0021 ; PubMed Central PMCID: PMC3342519. 22506599 PMC3342519

[31] Li H. Minimap2: pairwise alignment for nucleotide sequences. Bioinformatics. 2018;34(18):3094–100. Epub 2018/05/12. doi: 10.1093/bioinformatics/bty191 ; PubMed Central PMCID: PMC6137996. 29750242 PMC6137996

[32] Li H, Handsaker B, Wysoker A, Fennell T, Ruan J, Homer N, et al. The Sequence Alignment/Map format and SAMtools. Bioinformatics. 2009;25(16):2078–9. Epub 2009/06/10. doi: 10.1093/bioinformatics/btp352 ; PubMed Central PMCID: PMC2723002. 19505943 PMC2723002

[33] Altschul SF, Madden TL, Schaffer AA, Zhang J, Zhang Z, Miller W, et al. Gapped BLAST and PSI-BLAST: a new generation of protein database search programs. Nucleic Acids Res. 1997;25(17):3389–402. Epub 1997/09/01. doi: 10.1093/nar/25.17.3389 [pii]. ; PubMed Central PMCID: PMC146917. 9254694 PMC146917

[34] Benson DA, Karsch-Mizrachi I, Clark K, Lipman DJ, Ostell J, Sayers EW. GenBank. Nucleic Acids Res. 2012;40(Database issue):D48–53. Epub 2011/12/07. doi: 10.1093/nar/gkr1202 Epub 2011 Dec 5. ; PubMed Central PMCID: PMC3245039. 22144687 PMC3245039

[35] Gupta A, Jordan IK, Rishishwar L. stringMLST: a fast k-mer based tool for multilocus sequence typing. Bioinformatics. 2017;33(1):119–21. Epub 2016/09/09. doi: 10.1093/bioinformatics/btw586 . 27605103

[36] Seemann T. Prokka: rapid prokaryotic genome annotation. Bioinformatics. 2014;30(14):2068–9. doi: 10.1093/bioinformatics/btu153 Epub 2014 Mar 18. . 24642063

[37] Tonkin-Hill G, MacAlasdair N, Ruis C, Weimann A, Horesh G, Lees JA, et al. Producing polished prokaryotic pangenomes with the Panaroo pipeline. Genome Biol. 2020;21(1):180. Epub 2020/07/24. doi: 10.1186/s13059-020-02090-4 ; PubMed Central PMCID: PMC7376924. 32698896 PMC7376924

[38] Sahl JW, Caporaso JG, Rasko DA, Keim P. The large-scale blast score ratio (LS-BSR) pipeline: a method to rapidly compare genetic content between bacterial genomes. PeerJ 2014;2:e332. doi: 10.7717/peerj.332 24749011 PMC3976120

[39] Kent WJ. BLAT—the BLAST-like alignment tool. Genome research. 2002;12(4):656–64. Epub 2002/04/05. doi: 10.1101/gr.229202 Article published online before March 2002. ; PubMed Central PMCID: PMC187518. 11932250 PMC187518

[40] Rasko DA, Myers GS, Ravel J. Visualization of comparative genomic analyses by BLAST score ratio. BMC Bioinformatics. 2005;6:2. Epub 2005/01/07. doi: 10.1186/1471-2105-6-2 ; PubMed Central PMCID: PMC545078. 15634352 PMC545078

[41] Letunic I, Bork P. Interactive Tree Of Life (iTOL): an online tool for phylogenetic tree display and annotation. Bioinformatics. 2007;23(1):127–8. Epub 2006/10/20. doi: 10.1093/bioinformatics/btl529 . 17050570

[42] Delcher AL, Salzberg SL, Phillippy AM. Using MUMmer to identify similar regions in large sequence sets. Curr Protoc Bioinformatics. 2003;Chapter 10:Unit 10 3. Epub 2008/04/23. doi: 10.1002/0471250953.bi1003s00 18428693

[43] Sahl JW, Lemmer D, Travis J, Schupp JM, Gillece JD, Aziz M, et al. NASP: an accurate, rapid method for the identification of SNPs in WGS datasets that supports flexible input and output formats. Microb Genom. 2016;2(8):e000074. Epub 2017/03/30. doi: 10.1099/mgen.0.000074 ; PubMed Central PMCID: PMC5320593. 28348869 PMC5320593

[44] Holden MT, Titball RW, Peacock SJ, Cerdeno-Tarraga AM, Atkins T, Crossman LC, et al. Genomic plasticity of the causative agent of melioidosis, Burkholderia pseudomallei. Proc Natl Acad Sci U S A. 2004;101(39):14240–5. Epub 2004/09/21. doi: 10.1073/pnas.0403302101 ; PubMed Central PMCID: PMC521101. 15377794 PMC521101

[45] Price MN, Dehal PS, Arkin AP. FastTree 2—approximately maximum-likelihood trees for large alignments. PLoS One. 2010;5(3):e9490. Epub 2010/03/13. doi: 10.1371/journal.pone.0009490 ; PubMed Central PMCID: PMC2835736. 20224823 PMC2835736

[46] DePristo MA, Banks E, Poplin R, Garimella KV, Maguire JR, Hartl C, et al. A framework for variation discovery and genotyping using next-generation DNA sequencing data. Nat Genet. 2011;43(5):491–8. Epub 2011/04/12. doi: 10.1038/ng.806 ; PubMed Central PMCID: PMC3083463. 21478889 PMC3083463

[47] McKenna A, Hanna M, Banks E, Sivachenko A, Cibulskis K, Kernytsky A, et al. The Genome Analysis Toolkit: a MapReduce framework for analyzing next-generation DNA sequencing data. Genome research. 2010;20(9):1297–303. Epub 2010/07/21. doi: 10.1101/gr.107524.110 ; PubMed Central PMCID: PMC2928508. 20644199 PMC2928508

[48] Nguyen LT, Schmidt HA, von Haeseler A, Minh BQ. IQ-TREE: a fast and effective stochastic algorithm for estimating maximum-likelihood phylogenies. Mol Biol Evol. 2015;32(1):268–74. Epub 2014/11/06. doi: 10.1093/molbev/msu300 ; PubMed Central PMCID: PMC4271533. 25371430 PMC4271533

[49] Chernomor O, von Haeseler A, Minh BQ. Terrace Aware Data Structure for Phylogenomic Inference from Supermatrices. Syst Biol. 2016;65(6):997–1008. Epub 2016/04/29. doi: 10.1093/sysbio/syw037 ; PubMed Central PMCID: PMC5066062. 27121966 PMC5066062

[50] Schliep KP. phangorn: phylogenetic analysis in R. Bioinformatics. 2011;27(4):592–3. Epub 2010/12/21. doi: 10.1093/bioinformatics/btq706 ; PubMed Central PMCID: PMC3035803. 21169378 PMC3035803

[51] Cingolani P, Platts A, Wang le L, Coon M, Nguyen T, Wang L, et al. A program for annotating and predicting the effects of single nucleotide polymorphisms, SnpEff: SNPs in the genome of Drosophila melanogaster strain w1118; iso-2; iso-3. Fly (Austin). 2012;6(2):80–92. Epub 2012/06/26. doi: 10.4161/fly.19695 ; PubMed Central PMCID: PMC3679285. 22728672 PMC3679285

[52] Crispell J, Balaz D, Gordon SV. HomoplasyFinder: a simple tool to identify homoplasies on a phylogeny. Microb Genom. 2019;5(1). Epub 2019/01/22. doi: 10.1099/mgen.0.000245 ; PubMed Central PMCID: PMC6412054. 30663960 PMC6412054

[53] Raj A, Stephens M, Pritchard JK. fastSTRUCTURE: variational inference of population structure in large SNP data sets. Genetics. 2014;197(2):573–89. Epub 2014/04/05. doi: 10.1534/genetics.114.164350 ; PubMed Central PMCID: PMC4063916. 24700103 PMC4063916

[54] Croucher NJ, Page AJ, Connor TR, Delaney AJ, Keane JA, Bentley SD, et al. Rapid phylogenetic analysis of large samples of recombinant bacterial whole genome sequences using Gubbins. Nucleic Acids Res. 2015;43(3):e15. Epub 2014/11/22. doi: 10.1093/nar/gku1196 ; PubMed Central PMCID: PMC4330336. 25414349 PMC4330336

[55] Chewapreecha C, Holden MT, Vehkala M, Valimaki N, Yang Z, Harris SR, et al. Global and regional dissemination and evolution of Burkholderia pseudomallei. Nat Microbiol. 2017;2:16263. Epub 2017/01/24. doi: 10.1038/nmicrobiol.2016.263 ; PubMed Central PMCID: PMC5300093. 28112723 PMC5300093

[56] Drummond AJ, Rambaut A. BEAST: Bayesian evolutionary analysis by sampling trees. BMC Evol Biol. 2007;7:214. Epub 2007/11/13. doi: 10.1186/1471-2148-7-214 ; PubMed Central PMCID: PMC2247476. 17996036 PMC2247476

[57] Kumar S, Stecher G, Tamura K. MEGA7: Molecular Evolutionary Genetics Analysis Version 7.0 for Bigger Datasets. Mol Biol Evol. 2016;33(7):1870–4. Epub 2016/03/24. doi: 10.1093/molbev/msw054 . 27004904 PMC8210823

[58] Baele G, Lemey P, Bedford T, Rambaut A, Suchard MA, Alekseyenko AV. Improving the accuracy of demographic and molecular clock model comparison while accommodating phylogenetic uncertainty. Mol Biol Evol. 2012;29(9):2157–67. Epub 2012/03/10. doi: 10.1093/molbev/mss084 ; PubMed Central PMCID: PMC3424409. 22403239 PMC3424409

[59] Rambaut A, Drummond AJ, Xie D, Baele G, Suchard MA. Posterior Summarization in Bayesian Phylogenetics Using Tracer 1.7. Syst Biol. 2018;67(5):901–4. Epub 2018/05/03. doi: 10.1093/sysbio/syy032 ; PubMed Central PMCID: PMC6101584. 29718447 PMC6101584

[60] Barnes JL, Ketheesan N. Route of infection in melioidosis. Emerg Infect Dis. 2005;11(4):638–9. Epub 2005/04/20. doi: 10.3201/eid1104.041051 ; PubMed Central PMCID: PMC3320332. 15834987 PMC3320332

[61] Warawa JM. Evaluation of surrogate animal models of melioidosis. Front Microbiol. 2010;1:141. Epub 2010/01/01. doi: 10.3389/fmicb.2010.00141 ; PubMed Central PMCID: PMC3109346. 21772830 PMC3109346

[62] Li W, Godzik A. Cd-hit: a fast program for clustering and comparing large sets of protein or nucleotide sequences. Bioinformatics. 2006;22(13):1658–9. Epub 2006/05/30. doi: 10.1093/bioinformatics/btl158 . 16731699

[63] Lees JA, Galardini M, Bentley SD, Weiser JN, Corander J. pyseer: a comprehensive tool for microbial pangenome-wide association studies. Bioinformatics. 2018;34(24):4310–2. Epub 2018/12/12. doi: 10.1093/bioinformatics/bty539 ; PubMed Central PMCID: PMC6289128. 30535304 PMC6289128

[64] Edgar RC. MUSCLE: a multiple sequence alignment with reduced time and space complexity. BMC Bioinformatics. 2004;5:113–. doi: 10.1186/1471-2105-5-113 15318951 PMC517706

[65] Waterhouse AM, Procter JB, Martin DM, Clamp M, Barton GJ. Jalview Version 2—a multiple sequence alignment editor and analysis workbench. Bioinformatics. 2009;25(9):1189–91. Epub 2009/01/20. doi: 10.1093/bioinformatics/btp033 ; PubMed Central PMCID: PMC2672624. 19151095 PMC2672624

[66] Kursa MB, Jankowski A, Rudnicki WR. Boruta—A system for feature selection. Fundamenta Informaticae. 2010;101(4):271–85.

[67] Pedregosa F, Varoquaux G, Gramfort A, Michel V, Thirion B, Grisel O, et al. Scikit-learn: Machine Learning in Python. Journal of Machine Learning Research. 2011;12:2825–30.

[68] Sahl JW, Allender CJ, Colman RE, Califf KJ, Schupp JM, Currie BJ, et al. Genomic characterization of Burkholderia pseudomallei isolates selected for medical countermeasures testing: comparative genomics associated with differential virulence. PLoS One. 2015;10(3):e0121052. Epub 2015/03/25. doi: 10.1371/journal.pone.0121052 ; PubMed Central PMCID: PMC4372212. 25803742 PMC4372212

[69] Gertz EM, Yu YK, Agarwala R, Schaffer AA, Altschul SF. Composition-based statistics and translated nucleotide searches: improving the TBLASTN module of BLAST. BMC Biol. 2006;4:41. Epub 2006/12/13. doi: 10.1186/1741-7007-4-41 ; PubMed Central PMCID: PMC1779365. 17156431 PMC1779365

[70] De Smet B, Sarovich DS, Price EP, Mayo M, Theobald V, Kham C, et al. Whole-genome sequencing confirms that Burkholderia pseudomallei multilocus sequence types common to both Cambodia and Australia are due to homoplasy. J Clin Microbiol. 2015;53(1):323–6. Epub 2014/11/14. doi: 10.1128/JCM.02574-14 ; PubMed Central PMCID: PMC4290968. 25392354 PMC4290968

[71] Webb JR, Buller N, Rachlin A, Golledge C, Sarovich DS, Price EP, et al. A Persisting Nontropical Focus of Burkholderia pseudomallei with Limited Genome Evolution over Five Decades. mSystems. 2020;5(6). Epub 2020/11/12. doi: 10.1128/mSystems.00726-20 ; PubMed Central PMCID: PMC7657595. 33172968 PMC7657595

[72] Nguyen M, Brettin T, Long SW, Musser JM, Olsen RJ, Olson R, et al. Developing an in silico minimum inhibitory concentration panel test for Klebsiella pneumoniae. Sci Rep. 2018;8(1):421. Epub 2018/01/13. doi: 10.1038/s41598-017-18972-w ; PubMed Central PMCID: PMC5765115. 29323230 PMC5765115

[73] Nguyen M, Long SW, McDermott PF, Olsen RJ, Olson R, Stevens RL, et al. Using Machine Learning To Predict Antimicrobial MICs and Associated Genomic Features for Nontyphoidal Salmonella. J Clin Microbiol. 2019;57(2). Epub 2018/10/20. doi: 10.1128/JCM.01260-18 ; PubMed Central PMCID: PMC6355527. 30333126 PMC6355527

[74] Hu B, Li Y, Wang G, Zhang Y. The Blood Gene Expression Signature for Kawasaki Disease in Children Identified with Advanced Feature Selection Methods. Biomed Res Int. 2020;2020:6062436. Epub 2020/07/21. doi: 10.1155/2020/6062436 ; PubMed Central PMCID: PMC7327570 publication of this paper. 32685506 PMC7327570

[75] Reckseidler-Zenteno SL, Viteri DF, Moore R, Wong E, Tuanyok A, Woods DE. Characterization of the type III capsular polysaccharide produced by Burkholderia pseudomallei. J Med Microbiol. 2010;59(Pt 12):1403–14. Epub 2010/08/21. doi: 10.1099/jmm.0.022202-0 . 20724509

[76] Manzeniuk IN, Galina EA, Dorokhin VV, Kalachev I, Borzenkov VN, Svetoch EA. [Burkholderia mallei and Burkholderia pseudomallei. Study of immuno- and pathogenesis of glanders and melioidosis. Heterologous vaccines]. Antibiot Khimioter. 1999;44(6):21–6. Epub 1999/07/28. . 10422574

[77] Tan GG, Liu Y, Sivalingam SP, Sim SH, Wang D, Paucod JC, et al. Burkholderia pseudomallei aerosol infection results in differential inflammatory responses in BALB/c and C57Bl/6 mice. J Med Microbiol. 2008;57(Pt 4):508–15. Epub 2008/03/20. doi: 10.1099/jmm.0.47596-0 . 18349373

[78] Pearson T, Sahl JW, Hepp CM, Handady K, Hornstra H, Vazquez AJ, et al. Pathogen to commensal? Longitudinal within-host population dynamics, evolution, and adaptation during a chronic >16-year Burkholderia pseudomallei infection. PLoS Pathog. 2020;16(3):e1008298. Epub 2020/03/07. doi: 10.1371/journal.ppat.1008298 ; PubMed Central PMCID: PMC7077878. 32134991 PMC7077878

[79] Sahl JW, Pearson T, Okinaka R, Schupp JM, Gillece JD, Heaton H, et al. A Bacillus anthracis Genome Sequence from the Sverdlovsk 1979 Autopsy Specimens. mBio. 2016;7(5). Epub 2016/09/30. doi: 10.1128/mBio.01501-16 ; PubMed Central PMCID: PMC5050339. 27677796 PMC5050339

[80] Cui Y, Yu C, Yan Y, Li D, Li Y, Jombart T, et al. Historical variations in mutation rate in an epidemic pathogen, Yersinia pestis. Proc Natl Acad Sci U S A. 2013;110(2):577–82. Epub 2012/12/29. doi: 10.1073/pnas.1205750110 ; PubMed Central PMCID: PMC3545753. 23271803 PMC3545753

[81] Ohrman C, Sahl JW, Sjodin A, Uneklint I, Ballard R, Karlsson L, et al. Reorganized Genomic Taxonomy of Francisellaceae Enables Design of Robust Environmental PCR Assays for Detection of Francisella tularensis. Microorganisms. 2021;9(1). Epub 2021/01/15. doi: 10.3390/microorganisms9010146 ; PubMed Central PMCID: PMC7826819. 33440900 PMC7826819

[82] Keim P, Pearson T, Budowle B, Wilson M, Wagner DM. Chapter 25—Microbial forensic investigations in the context of bacterial population genetics. In: Budowle B, Schutzer S, Morse S, editors. Microbial Forensics (Third Edition): Academic Press; 2020. p. 381–92.

[83] Limmathurotsakul D, Golding N, Dance DA, Messina JP, Pigott DM, Moyes CL, et al. Predicted global distribution of Burkholderia pseudomallei and burden of melioidosis. Nat Microbiol. 2016;1(1). Epub 2016/02/16. doi: 10.1038/nmicrobiol.2015.8 ; PubMed Central PMCID: PMC4746747. 26877885 PMC4746747

