# Supplementary figures and images for: Multiple phylogenetically-diverse, differentially-virulent Burkholderia pseudomallei isolated from a single soil sample collected in Thailand
Source: PLoS Negl Trop Dis. 2022 Feb 10;16(2):e0010172. doi: 10.1371/journal.pntd.0010172 (PMC8865643; doi:10.1371/journal.pntd.0010172)

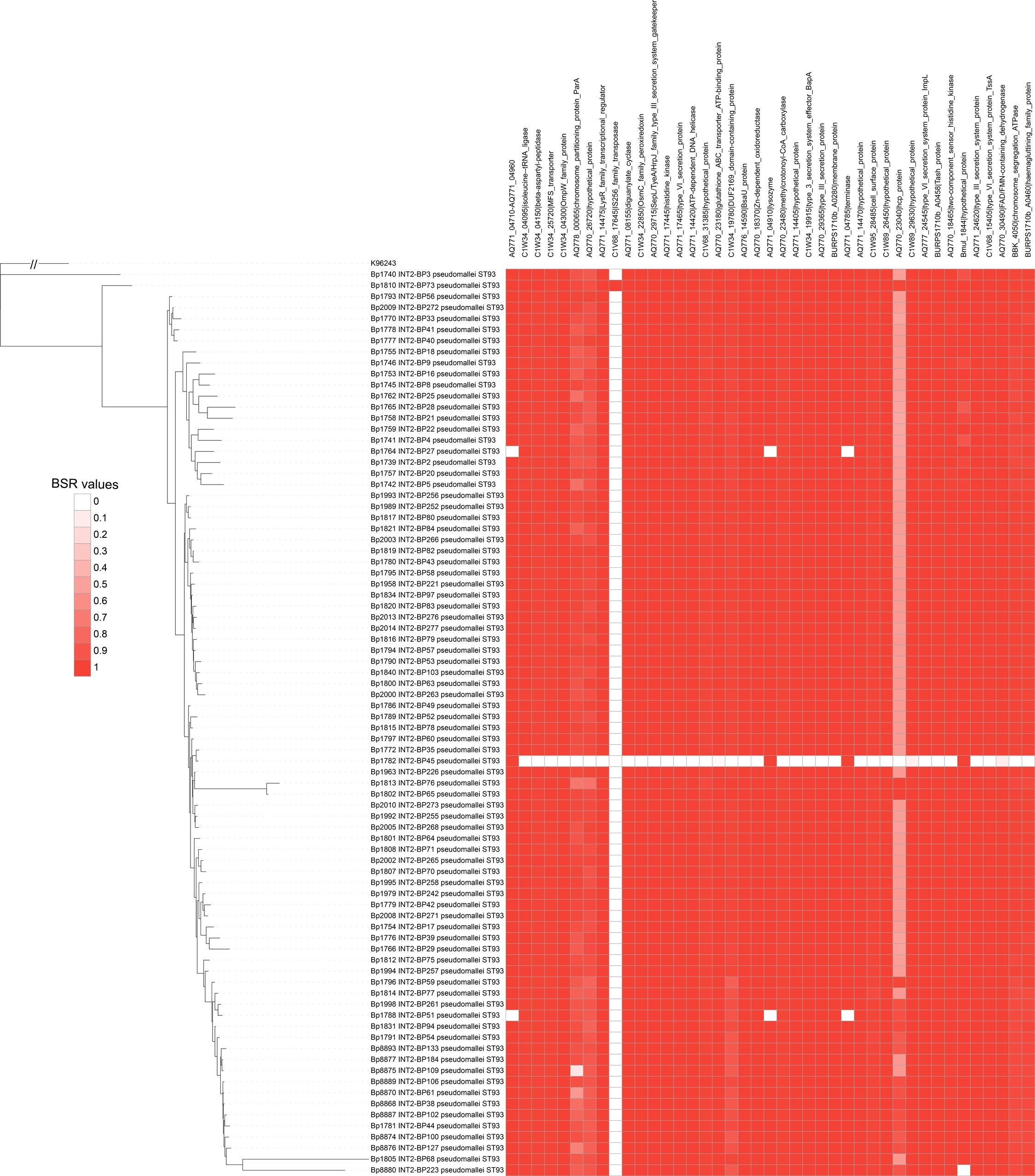

Supplement: S1 Fig — The phylogeny was annotated with coding regions that show variable distribution, based on an analysis with LS-BSR [38]. The phylogeny and heatmap were visualized with the interactive tree of life [41] and rooted with B. pseudomallei K96243 [44] as it represents an outgroup genome from Thailand. (TIF) [file pntd.0010172.s001.tif]

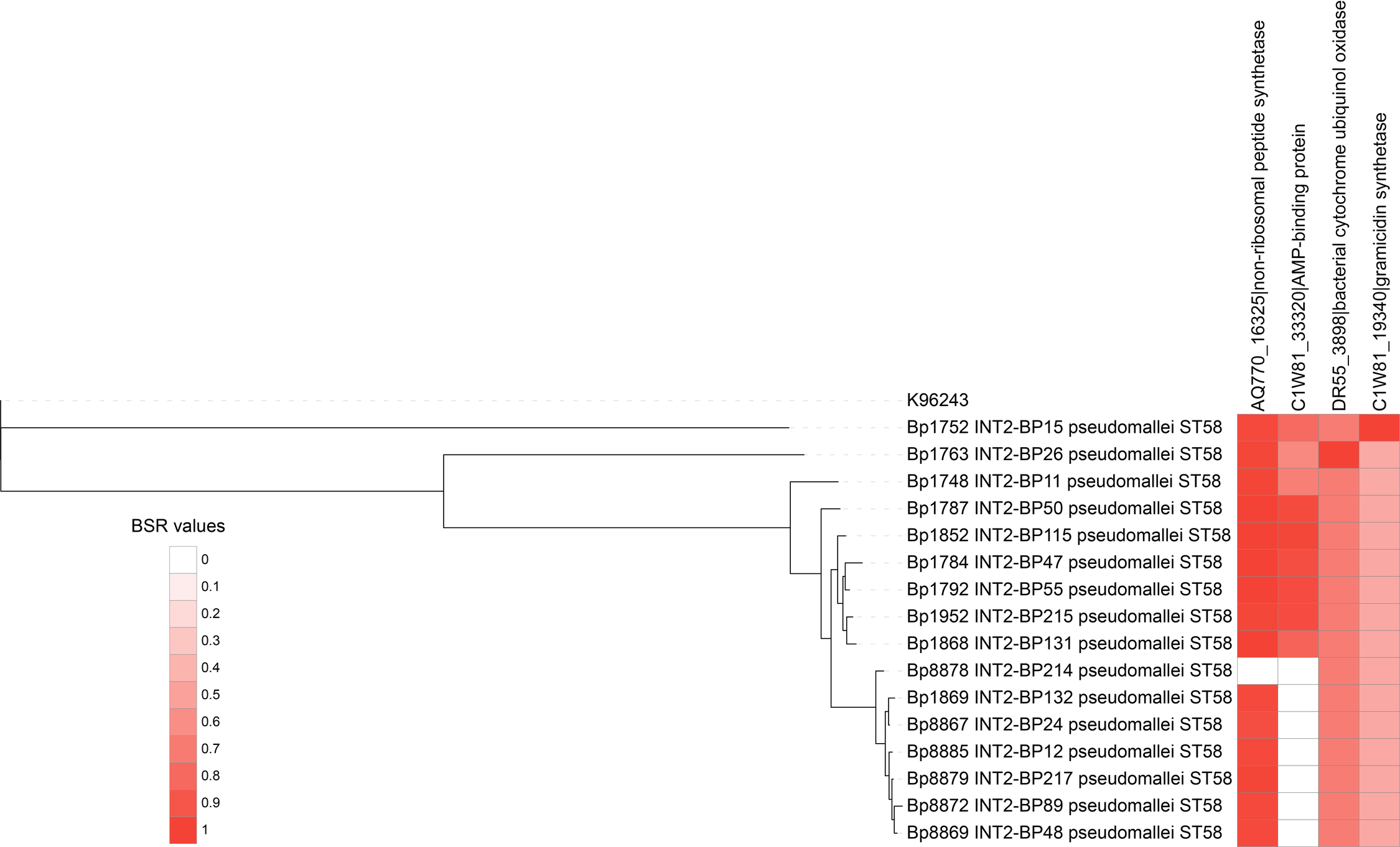

Supplement: S2 Fig — The phylogeny was annotated with coding regions that show variable distribution, based on an analysis with LS-BSR [38]. The phylogeny and heatmap were visualized with the interactive tree of life [41] and rooted with B. pseudomallei K96243 [44] as it represents an outgroup genome from Thailand. (TIF) [file pntd.0010172.s002.tif]

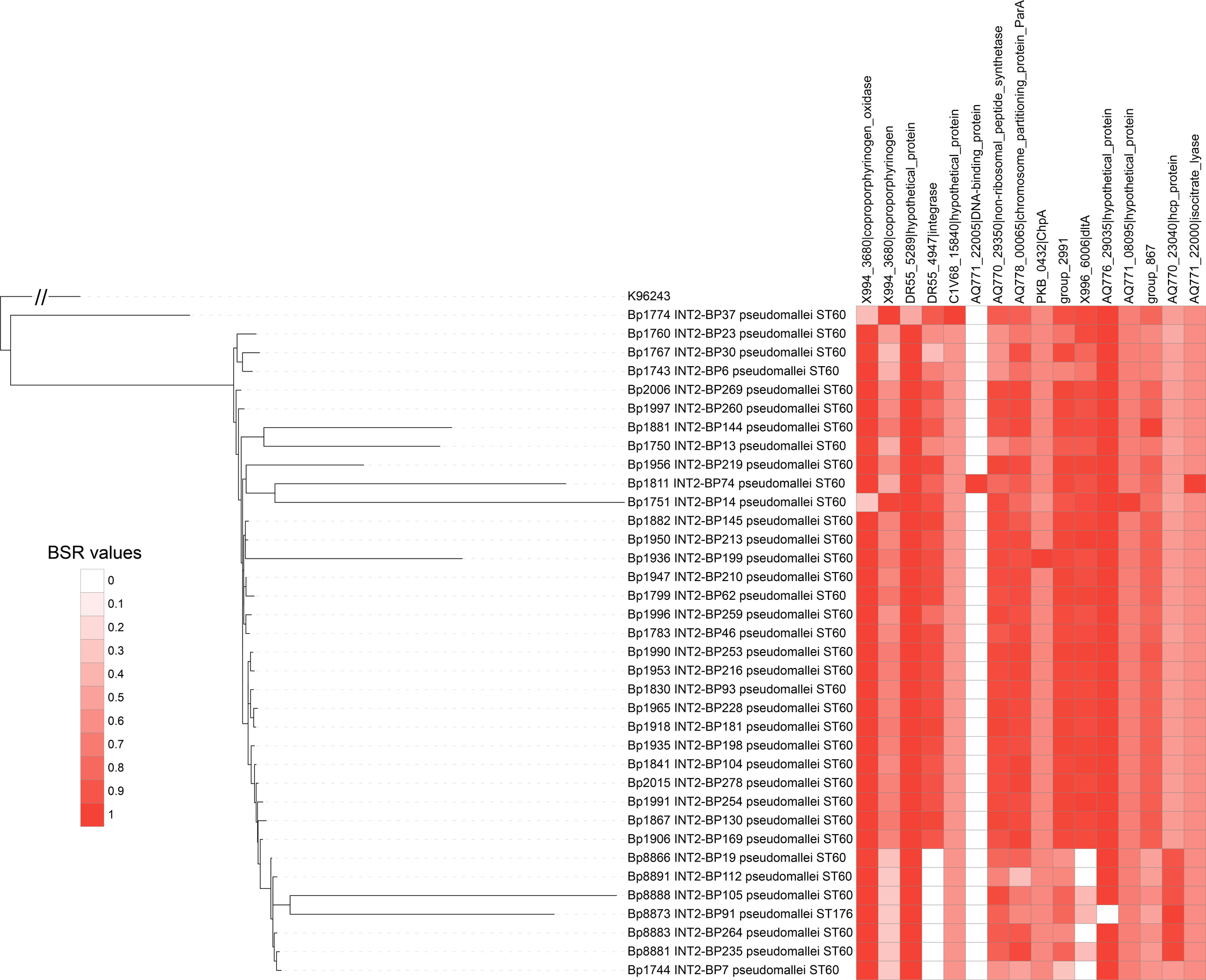

Supplement: S3 Fig — The phylogeny was annotated with coding regions that show variable distribution, based on an analysis with LS-BSR [38]. The phylogeny and heatmap were visualized with the interactive tree of life [41] and rooted with B. pseudomallei K96243 [44] as it represents an outgroup genome from Thailand. (TIF) [file pntd.0010172.s003.tif]

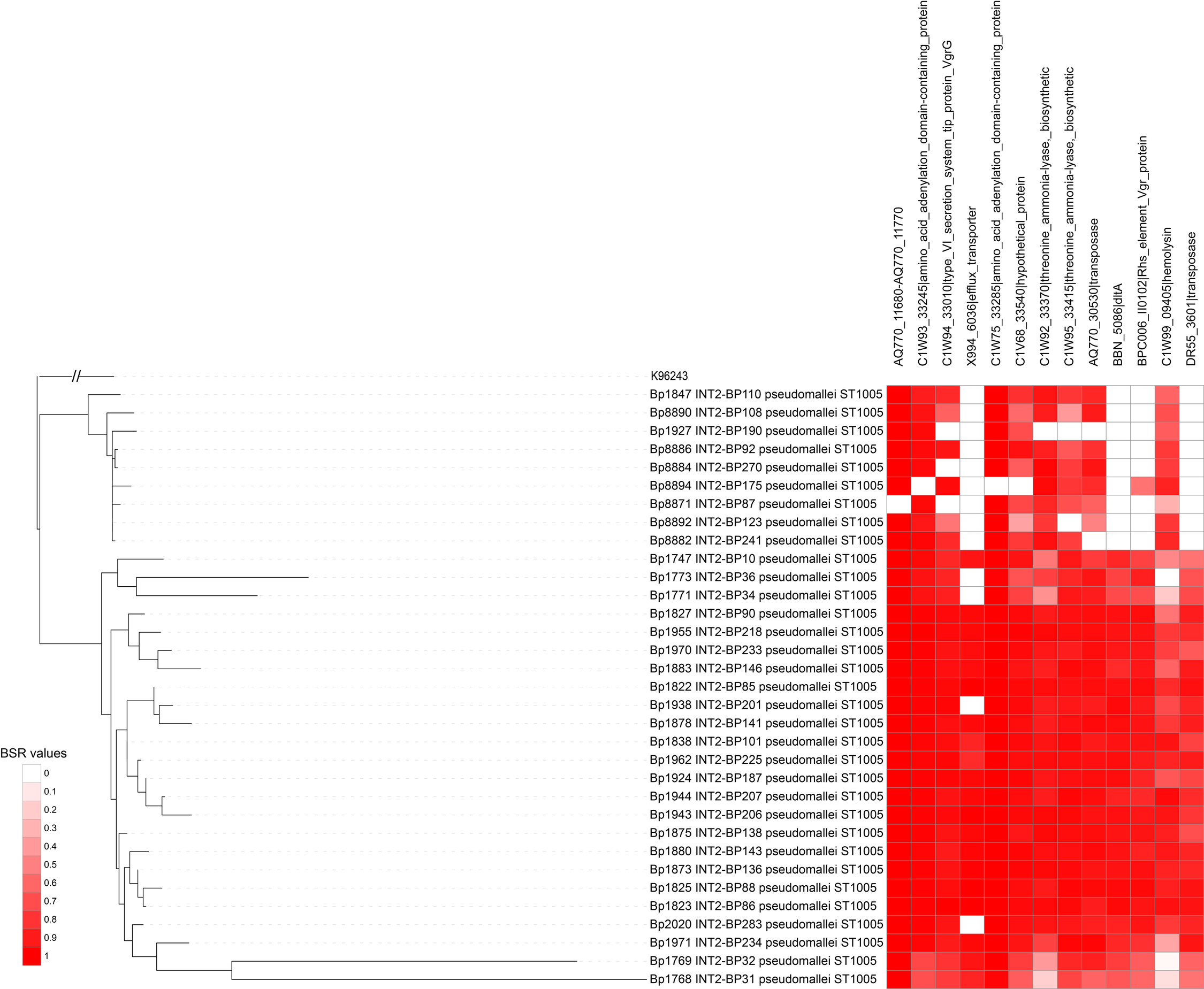

Supplement: S4 Fig — The phylogeny was annotated with coding regions that show variable distribution, based on an analysis with LS-BSR [38] using the TVMe+ASC+R3 substitution model [49]. The phylogeny and heatmap were visualized with the interactive tree of life [41] and rooted with B. pseudomallei K96243 [44] as it represents an outgroup genome from Thailand. (TIF) [file pntd.0010172.s004.tif]

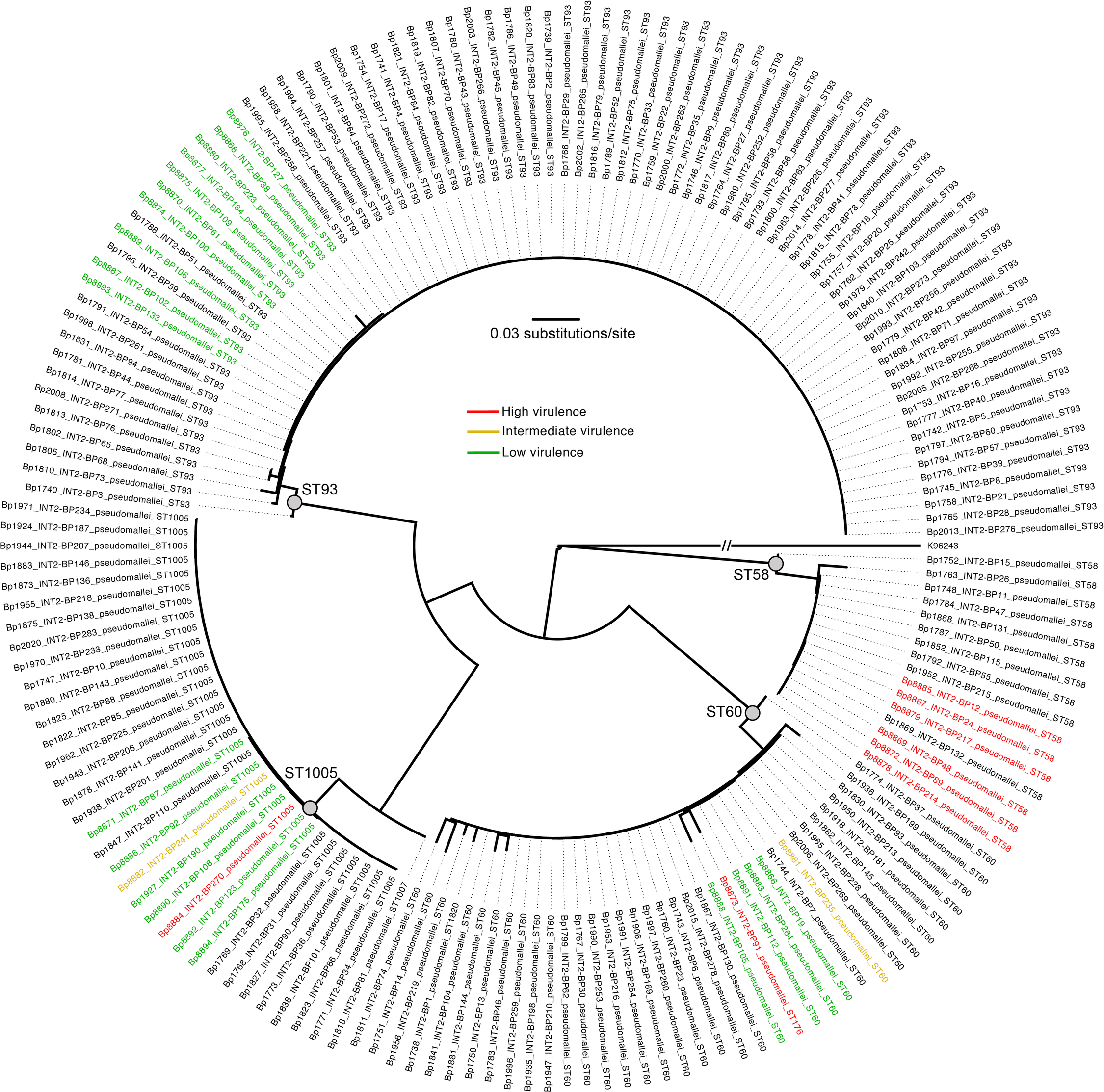

Supplement: S5 Fig — Animal-passed genomes are colored by attenuated, intermediate, or virulent phenotypes. The phylogeny is rooted by B. pseudomallei K96243 [44] as it represents an outgroup genome from Thailand. (TIF) [file pntd.0010172.s005.tif]

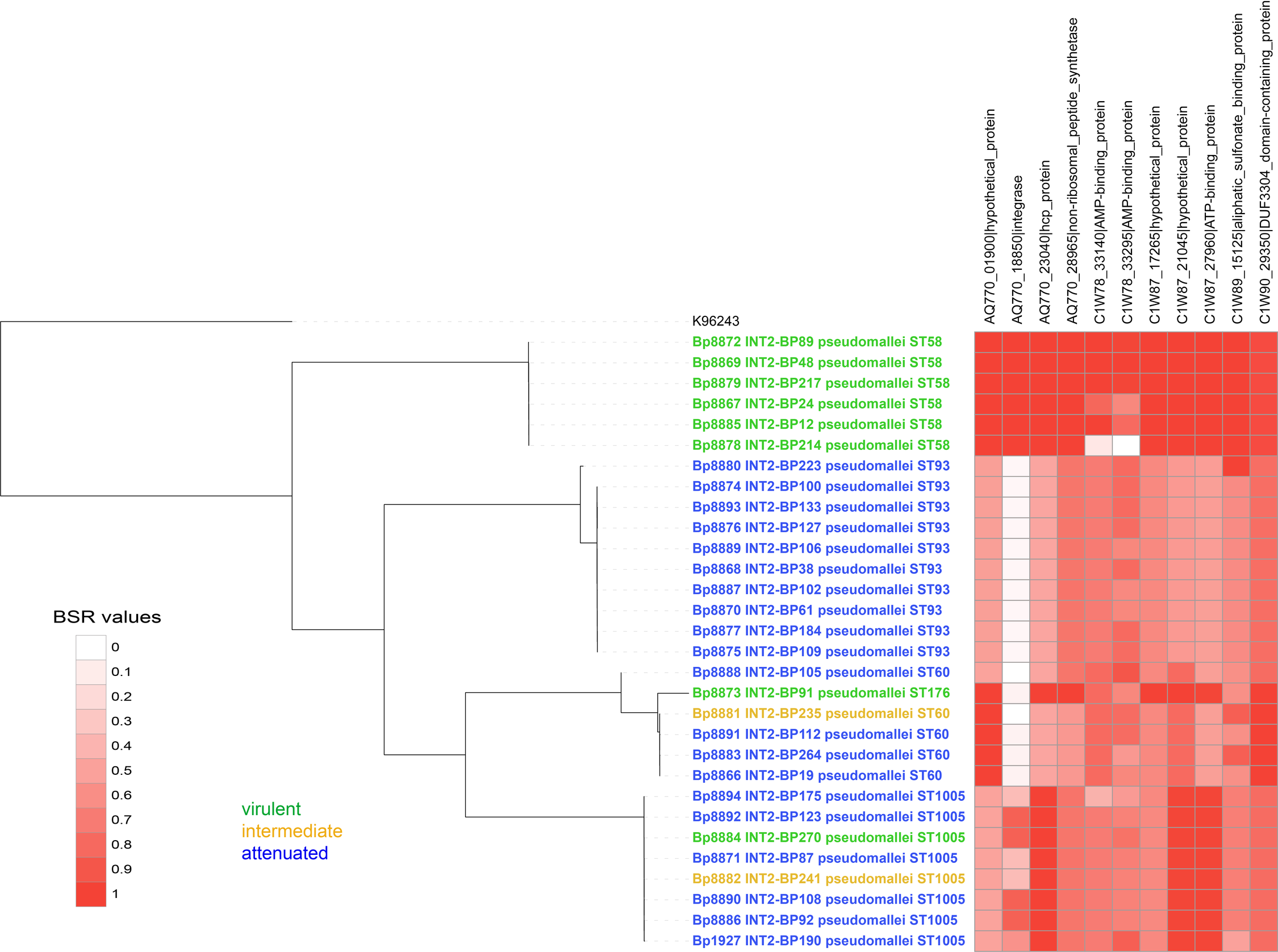

Supplement: S6 Fig — Genomes are colored by the virulence outcome in an animal challenge model. Each genome was screened with LS-BSR [38] using genes identified through machine learning methods. The phylogeny and heatmap were visualized with the interactive tree of life [41] and rooted with B. pseudomallei K96243 [44] as it represents an outgroup genome from Thailand. (TIF) [file pntd.0010172.s006.tif]

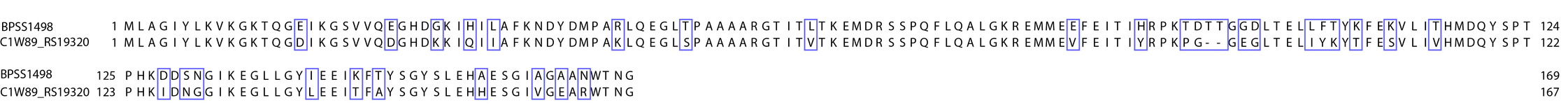

Supplement: S7 Fig — Blue boxes surround amino acid differences in the alignment, which was visualized with Jalview [65]. (TIF) [file pntd.0010172.s007.tif]

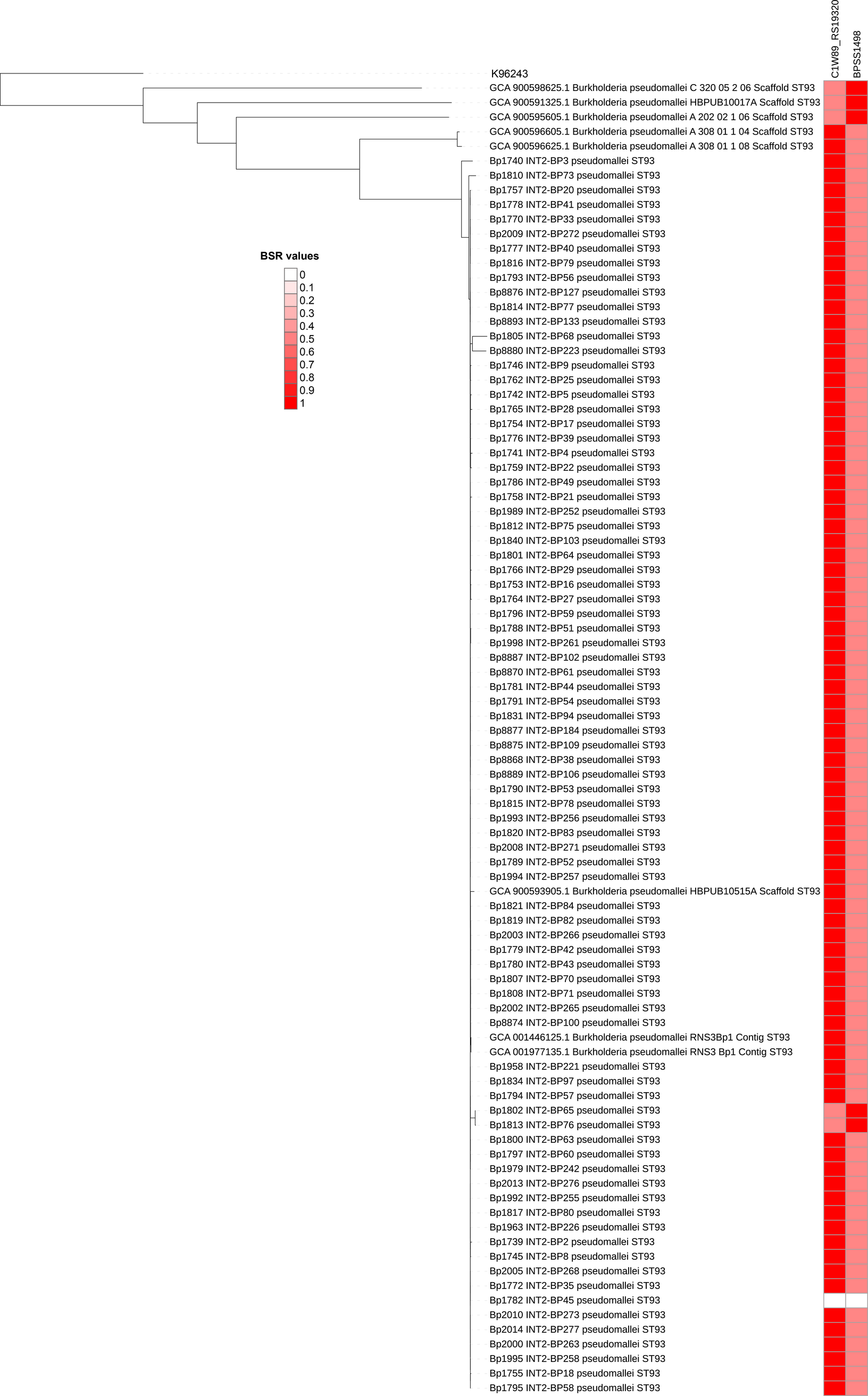

Supplement: S8 Fig — The phylogeny was rooted with B. pseudomallei K96243 [44]. Each genome was screened with LS-BSR [38] using two Hcp1 variants. The phylogeny and heatmap were visualized with the interactive tree of life [41] and rooted with B. pseudomallei K96243 [44] as it represents an outgroup genome from Thailand. (TIF) [file pntd.0010172.s008.tif]
