# Supplementary material for: Multiple phylogenetically-diverse, differentially-virulent Burkholderia pseudomallei isolated from a single soil sample collected in Thailand
Source: PLoS Negl Trop Dis. 2022 Feb 10;16(2):e0010172. doi: 10.1371/journal.pntd.0010172 (PMC8865643; doi:10.1371/journal.pntd.0010172)
Supplement: Attachment — Submitted filename: PLOSNTD_comments.pdf [file pntd.0010172.s013.pdf]

The majority of the revisions made by the authors have greatly improved the quality and clarity of this manuscript. However, there are a few sections that require further discussion and/or modification prior to publication. Given the comments below, this reviewer considers them major revisions but also believes that the revisions are achievable by the authors.

1. Animal model:

The addition of controls and references has greatly improved this section. For the purposes of this publication, time-to-death is acceptable as an initial readout of differential virulence provided the authors clearly indicate within the manuscript the limitations of their interpretation in the absence of organ counts, basic histology and /or LD_{50} determinations. The paper cited by the authors is from 2005 and provides a 10-day LD_{50} for SC inoculation with strain NCTC13178 in Balb/*c* mice. The authors did use the same strain as a control, and this does provide a reasonable starting point for studies. However, as mechanisms of virulence and attenuation can very between strains and hosts, determination of 50% infection or lethal doses along with bacterial counts would give a much better indication of differential virulence as illustrated in a more recent study from the same group (Morris et al., Emerg Infect Dis. 2015 Jan;21(1):58-63). In the absence of basic histology/organ counts the authors should acknowledge that bacterial colonization, dissemination, tissue tropism and other factors that lead to illness or death may considerably between strains and could reflect different mechanisms that result in the data observed. For example, attenuation in some cases may still result in colonization…in other cases this may not be the case. Furthermore, if the dose for inoculation is well below an ID/LD_{50} one must be cautious in describing the observed results as translation to differential virulence or even attenuation in humans as strains that did not result in death in this study could still be very lethal. This is a reason why high doses of organisms are used in protection studies as it is not uncommon to see all mice survive if the inoculation dose is not titrated correctly. And also why it is essential to minimally characterize bacterial counts in all animals at the end of the study.

In the absence of titration data for ID/LD_{50} values, bacterial counts and/or histology, there is simply not enough evidence to support the description of the results as indicative of differential virulence – where virulence is generally understood as the capacity to cause disease in a host. What this study does support is that the strains studied may indeed have different levels of infectious doses that results in different lethality numbers over the period of the animal study – and this is worth publishing, The authors could potentially argue that their data may suggest that differences in infectious doses could result in variations of disease presentation in humans (but not necessarily a lack of lethality) – though the amount of organism required to cause disease in humans is also highly dependent on risk factors (age, diabetes, and other conditions that can result in being immunocompromised).

2. Machine Learning

With all due respect to the authors, this reviewer is struggling to understand exactly what input were used to undertake the ML predictions. The methodology indicates a "LS-BSR matrix" but no other information is provided so it is difficult to know how the ML is being used here to identify patterns/correlations. How is the random forest

classifier being used in this model? Are there gaps in the data? I understand that its use will reduce overfitting but what is the training set that being used? Is the LS-BSR matrix generated exclusively using sequences described in this manuscript? If so, then it is possible that the results generated are heavily biased and this may explain my concerns about the identification of sequence variation in Hcp1 as being linked to observed results from the animal model studies.

Indeed, the authors themselves note that the ML Boruta model has not been previously used for bacterial GWAS and this reviewer suspects there may be a reason for this. These authors were previously asked to consider benchmarking the use of this method using established sequence data that is known to impact on the virulence properties of B. pseudomallei. The justification provided for using this ML approach "to identify genes associated with virulence that were not obvious by other methods" is a weak statement as the meaning of "not obvious" needs to be clearly defined by the authors using concrete evidence from their data analyses that led to this decision. My concern is that in absence of benchmarking, that the correlations shown in Figure 6 may be very weak or artefacts.

ML can be very useful, and I am quite supportive of its application as an exploratory method, but the results obtained require more analysis then just providing a list of sequences on a novel dataset to support the use of a previously untested methodology in analysis of bacterial sequences. If the authors still wish to publish these data then there needs to be more discussion about the risks of the approach and the need for benchmarking and better validation.

3. Hcp1 sequence analysis and interpretation

The authors make the following statements in this manuscript:

"However, 14 of 20 attenuated isolates, plus one intermediate virulence isolate (Bp8881), contain a variant of Hcp1 (C1W89_RS19320) with significant amino acid differences to BPSS1498 (Figure S7). This demonstrates that this Hcp1 homolog has a different peptide composition and may have a different protein function associated with attenuated virulence."

"Of external ST93 genome assemblies, 3 have the K96243 version of Hcp1, while 5 have the alternate allele; one of the external genomes with the alternate Hcp1 allele was associated with humans (SAMN04208633), although details of the isolation source are not available in the public record."

And in their reviewer responses they also state:

"We have changed the language to reflect that the change may be altered and not very different. MUSCLE was used to align amino acid sequences and the citation and version are in the original manuscript. There are no statistics, as even a single amino acid difference would constitute a different protein sequence. There is a wealth of literature in B. pseudomallei and other bacterial and viral pathogens to demonstrate that a single

amino acid difference can drastically affect a phenotype, which was the point that we were making."

It is evident from the results obtained in the animal model studies and that the authors are trying to find an explanation for the observed results using sequence analysis techniques and this is understandable. However, this reviewer does not support the interpretation of the Hcp1 variant and considers it to be flawed for the following reasons:

1. Although the authors' justification for suggesting that the Hcp1 sequence variant may have a different function based on its composition, this is an extreme interpretation compared to prevailing knowledge about protein-structure function relationships, protein evolution and the identification of homologs within and across species boundaries. For example, within a single bacterial species, full genome sequencing of different strains and subsequent translation of encoded gene products demonstrate single (or more) residue changes in the majority of encoded proteins without a loss or gain of function.
2. The bioinformatic analyses undertaken is incomplete and lacks rigor. A standard and basic analysis of the variant sequence using the BlastP suite (any of the options) against the non-redundant protein sequence database shows that this sequence aligns with at least 100 annotated sequences for Hcp1/Hcp Type IV effector family protein with many bacterial species – with tops hits being B. pseudomallei.

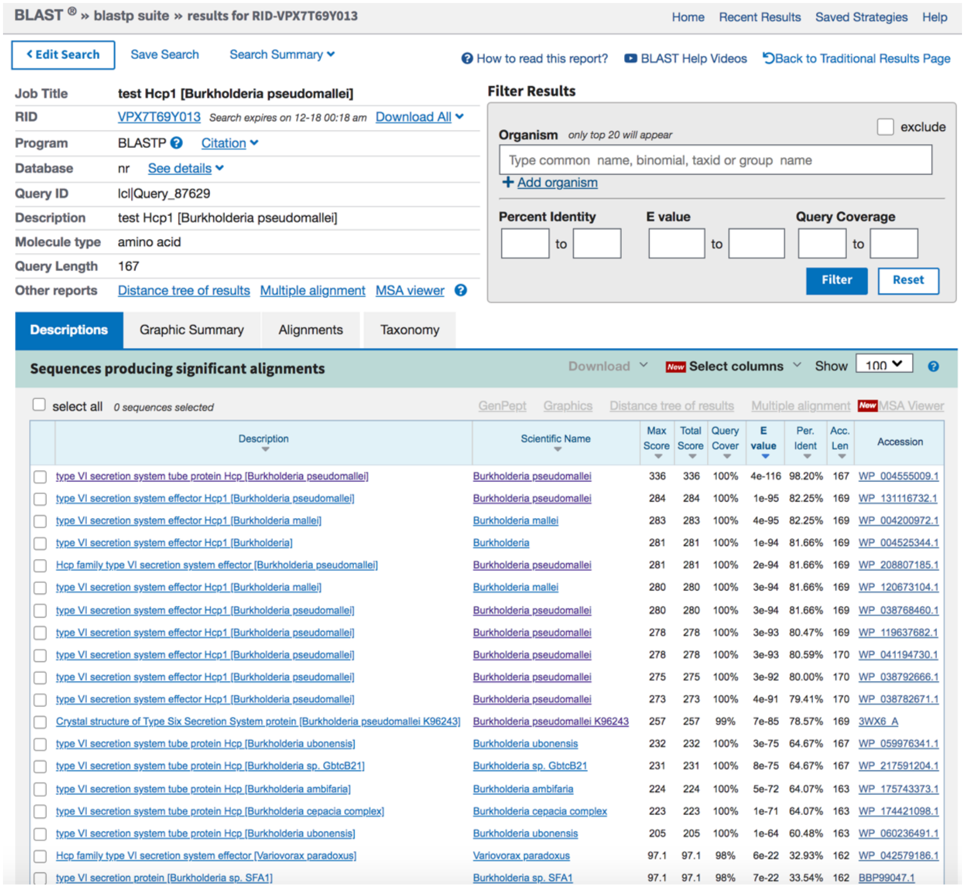

BLAST ® » blastp suite » results for RID-VPX7T69Y013

Home Recent Results Saved Strategies Help

‹ Edit Search | Save Search | Search Summary

How to read this report? | BLAST Help Videos | Back to Traditional Results Page

Job Title	test Hcp1 [Burkholderia pseudomallei]
RID	VPX7T69Y013 Search expires on 12-18 00:18 am Download All
Program	BLASTP Citation
Database	nr See details
Query ID	lcl|Query_87629
Description	test Hcp1 [Burkholderia pseudomallei]
Molecule type	amino acid
Query Length	167
Other reports	Distance tree of results Multiple alignment MSA viewer

Filter Results

Organism only top 20 will appear — exclude

Type common name, binomial, taxid or group name

+ Add organism

Percent Identity ___ to ___ | E value ___ to ___ | Query Coverage ___ to ___

Filter Reset

Descriptions | Graphic Summary | Alignments | Taxonomy

Sequences producing significant alignments

Download | New Select columns | Show 100

select all 0 sequences selected

GenPept Graphics Distance tree of results Multiple alignment New MSA Viewer

Description	Scientific Name	Max Score	Total Score	Query Cover	E value	Per. Ident	Acc. Len	Accession
type VI secretion system tube protein Hcp [Burkholderia pseudomallei]	Burkholderia pseudomallei	336	336	100%	4e-116	98.20%	167	WP_004555009.1
type VI secretion system effector Hcp1 [Burkholderia pseudomallei]	Burkholderia pseudomallei	284	284	100%	1e-95	82.25%	169	WP_131116732.1
type VI secretion system effector Hcp1 [Burkholderia mallei]	Burkholderia mallei	283	283	100%	4e-95	82.25%	169	WP_004200972.1
type VI secretion system effector Hcp1 [Burkholderia]	Burkholderia	281	281	100%	1e-94	81.66%	169	WP_004525344.1
Hcp family type VI secretion system effector [Burkholderia pseudomallei]	Burkholderia pseudomallei	281	281	100%	2e-94	81.66%	169	WP_208807185.1
type VI secretion system effector Hcp1 [Burkholderia mallei]	Burkholderia mallei	280	280	100%	3e-94	81.66%	169	WP_120673104.1
type VI secretion system effector Hcp1 [Burkholderia pseudomallei]	Burkholderia pseudomallei	280	280	100%	3e-94	81.66%	169	WP_038768460.1
type VI secretion system effector Hcp1 [Burkholderia pseudomallei]	Burkholderia pseudomallei	278	278	100%	3e-93	80.47%	169	WP_119637682.1
type VI secretion system effector Hcp1 [Burkholderia pseudomallei]	Burkholderia pseudomallei	278	278	100%	3e-93	80.59%	170	WP_041194730.1
type VI secretion system effector Hcp1 [Burkholderia pseudomallei]	Burkholderia pseudomallei	275	275	100%	3e-92	80.00%	170	WP_038792666.1
type VI secretion system effector Hcp1 [Burkholderia pseudomallei]	Burkholderia pseudomallei	273	273	100%	4e-91	79.41%	170	WP_038782671.1
Crystal structure of Type Six Secretion System protein [Burkholderia pseudomallei K96243]	Burkholderia pseudomallei K96243	257	257	99%	7e-85	78.57%	169	3WX6_A
type VI secretion system tube protein Hcp [Burkholderia ubonensis]	Burkholderia ubonensis	232	232	100%	3e-75	64.67%	167	WP_059976341.1
type VI secretion system tube protein Hcp [Burkholderia sp. GbtcB21]	Burkholderia sp. GbtcB21	231	231	100%	8e-75	64.67%	167	WP_217591204.1
type VI secretion system tube protein Hcp [Burkholderia ambifaria]	Burkholderia ambifaria	224	224	100%	5e-72	64.07%	163	WP_175743373.1
type VI secretion system tube protein Hcp [Burkholderia cepacia complex]	Burkholderia cepacia complex	223	223	100%	1e-71	64.07%	163	WP_174421098.1
type VI secretion system tube protein Hcp [Burkholderia ubonensis]	Burkholderia ubonensis	205	205	100%	1e-64	60.48%	163	WP_060236491.1
Hcp family type VI secretion system effector [Variovorax paradoxus]	Variovorax paradoxus	97.1	97.1	98%	6e-22	32.93%	162	WP_042579186.1
type VI secretion protein [Burkholderia sp. SFA1]	Burkholderia sp. SFA1	97.1	97.1	98%	7e-22	33.54%	162	BBP99047.1

3. The top hit is for a B. pseudomallei sequence WP_004555009.1.

```
LOCUS       WP_004555009              167 aa            linear   BCT 19-JUN-
2019
DEFINITION  type VI secretion system tube protein Hcp [Burkholderia
            pseudomallei].
ACCESSION   WP_004555009
VERSION     WP_004555009.1
KEYWORDS    RefSeq.
SOURCE      Burkholderia pseudomallei
  ORGANISM  Burkholderia pseudomallei
            Bacteria; Proteobacteria; Betaproteobacteria; Burkholderiales;
            Burkholderiaceae; Burkholderia; pseudomallei group.
REFERENCE   1  (residues 1 to 167)
  AUTHORS   Mougous,J.D., Cuff,M.E., Raunser,S., Shen,A., Zhou,M.,
            Gifford,C.A., Goodman,A.L., Joachimiak,G., Ordonez,C.L.,
Lory,S.,
            Walz,T., Joachimiak,A. and Mekalanos,J.J.
  TITLE     A virulence locus of Pseudomonas aeruginosa encodes a protein
            secretion apparatus
  JOURNAL   Science 312 (5779), 1526-1530 (2006)
   PUBMED   16763151
COMMENT     REFSEQ: This record represents a single, non-redundant, protein
            sequence which may be annotated on many different RefSeq
genomes
            from the same, or different, species.

            ##Evidence-For-Name-Assignment-START##
            Evidence Category  :: HMM
            Evidence Accession :: TIGR03344.1
            Evidence Source    :: JCVI
            Source Identifier  :: TIGR03344
            ##Evidence-For-Name-Assignment-END##
            COMPLETENESS: full length.
FEATURES             Location/Qualifiers
     source          1..167
                     /organism="Burkholderia pseudomallei"
                     /db_xref="taxon:28450"
     gene            1..167
                     /gene="hcp"
     Protein         1..167
                     /product="type VI secretion system tube protein Hcp"
                     /calculated_mol_wt=18411
     Region          5..167
                     /region_name="T6SS_HCP"
                     /note="Type VI secretion system effector, Hcp;
cl01226"
                     /db_xref="CDD:321401"
ORIGIN
        1 mlagiylkvk gktqgdikgs vvqdghdkki qiiafkndyd mpaklqegls paaaargtit
       61 vtkemdrssp qflqalgkre mmevfeitiy rpkpggeglt eliykytfes vlivhmdqys
      121 ptphkidngg ikegllgyle eitfaysgys lehhesgivg earwtng
//
```

The alignment with the Hcp1 sequence is shown below. It has an identity of 98% with three amino acid changes – a conservative substitution and an inversion. A skilled protein bioinformaticist would not consider these changes likely to lead to any gain or loss of function.

4. The unlikeliness of this sequence functioning in any way other than as annotated as Hcp1 is further supported by the identification of the identical sequence in 376 entries in the NCBI (see below). The strains listed have shown this sequence to be identified in isolates from clinical strains and environmental samples, some of which contain information about the location and source of isolation.

Identical Protein Groups ▾ Send to: ▾

type VI secretion system tube protein Hcp

GenPept FASTA Graphics BLAST

Name: type VI secretion system tube protein Hcp
RefSeq Selected Product: WP_004555009.1, 167 amino acids
Taxonomic Group: b-proteobacteria
Assembly Accessions: 376
Protein Accessions: 187
CDS Regions: 376
Total Rows: 376

Protein Classes
☑ WP_004555009.1 - 190 rows
☑ INSDC - 186 rows
Update Report

<<First <Prev Page 1 of 8 (50 CDSs per page) Next > Last>>

Source (All)	CDS Region in Nucleotide	Protein	Name	Organism	Strain	Assembly
RefSeq	NZ_AHJB01000260.1 7165-7668 (+)	WP_004555009.1	type VI secretion system tube protein Hcp	Burkholderia pseudomallei 1258a	1258a	GCF_000259755.1
RefSeq	NZ_AHJC01000281.1 7167-7670 (+)	WP_004555009.1	type VI secretion system tube protein Hcp	Burkholderia pseudomallei 1258b	1258b	GCF_000259775.1
RefSeq	NZ_ARZU01000166.1 38301-38804 (-)	WP_004555009.1	type VI secretion system tube protein Hcp	Burkholderia pseudomallei RNS3Bp1	RNS3Bp1	GCF_001446125.1
RefSeq	NZ_ARZV01000150.1 21365-21868 (+)	WP_004555009.1	type VI secretion system tube protein Hcp	Burkholderia pseudomallei RNS7Bp6	RNS7Bp6	GCF_001446155.1
RefSeq	NZ_CGFC01000014.1 21249-21752 (+)	WP_004555009.1	type VI secretion system tube protein Hcp	Burkholderia pseudomallei	E8	GCF_001327435.1
RefSeq	NZ_CHFI01000005.1 190200-190703 (+)	WP_004555009.1	type VI secretion system tube protein Hcp	Burkholderia pseudomallei	E0387	GCF_001327555.1
RefSeq	NZ_JAAAAC010000024.1 21346-21849 (+)	WP_004555009.1	type VI secretion system tube protein Hcp	Burkholderia pseudomallei	Bp1774	GCF_009904095.1
RefSeq	NZ_JAAAAE010000057.1 21400-21903 (+)	WP_004555009.1	type VI secretion system tube protein Hcp	Burkholderia pseudomallei	Bp1810	GCF_009904185.1
RefSeq	NZ_JAAAAF010000014.1 160487-160990 (-)	WP_004555009.1	type VI secretion system tube protein Hcp	Burkholderia pseudomallei	Bp8880	GCF_009904165.1
RefSeq	NZ_JAAAAG010000008.1 160482-160985 (-)	WP_004555009.1	type VI secretion system tube protein Hcp	Burkholderia pseudomallei	Bp8870	GCF_009904175.1
RefSeq	NZ_JAAAAH010000013.1 172962-173465 (-)	WP_004555009.1	type VI secretion system tube protein [illegible]	Burkholderia pseudomallei	Bp8887	GCF_009904215.1

Given the wealth of information available (and this analysis could be further extended if needed), the case for suggesting that this Hcp1 variant sequence "has a different peptide composition and may have a different protein function associated with attenuated virulence" – is not adequately made – taking into account the animal experiments and high

sequence identity of this variant to a nearly identical sequence present in many virulent B. pseudomallei strains. Choosing to use MUSCLE to align the sequence with the sequence from Hcp1 sequence from the K96243 strain gives a biased and incomplete analysis given the large amount of sequencing data available and the ease with which to conduct searches usually publicly available software that is considered reliable by most of the molecular biology community. It is not difficult to quote statistics for alignments (including identity and similarity percentages). Protein bioinformaticists typically carry out alignments and searches using several different software to take into account different underlying biasing in alignment algorithms. Finally, the suggestion that there is a wealth of literature in B. pseudomallei and other pathogens to demonstrate that a single amino acid change can change a phenotype is true. However, the virulence of B. pseudomallei has always been considered multi-factorial (not generally reliant upon a single factor like a toxin). It would be an interesting analysis to cite the published studies of naturally occurring strains of B. pseudomallei where a single residue change has been directly ascribed to a change in phenotype (other than a truncation of loss of protein) and to include those references in the discussion as they would be very valuable.

In summary, this reviewer does consider this paper worthy of publication but with a careful re-analysis of the data and the methods used, and a more restrained interpretation of the results.
