# Supplementary material for: Multiple phylogenetically-diverse, differentially-virulent Burkholderia pseudomallei isolated from a single soil sample collected in Thailand
Source: PLoS Negl Trop Dis. 2022 Feb 10;16(2):e0010172. doi: 10.1371/journal.pntd.0010172 (PMC8865643; doi:10.1371/journal.pntd.0010172)
Supplement: Attachment — Submitted filename: response_to_reviewers_V2.docx [file pntd.0010172.s014.docx]

The majority of the revisions made by the authors have greatly improved the quality and

clarity of this manuscript. However, there are a few sections that require further discussion

and/or modification prior to publication. Given the comments below, this reviewer

considers them major revisions but also believes that the revisions are achievable by the

authors.

1. Animal model:

The addition of controls and references has greatly improved this section. For the

purposes of this publication, time-to-death is acceptable as an initial readout of

differential virulence provided the authors clearly indicate within the manuscript the

limitations of their interpretation in the absence of organ counts, basic histology and /or

LD50 determinations. The paper cited by the authors is from 2005 and provides a 10-day

LD50 for SC inoculation with strain NCTC13178 in Balb/c mice. The authors did use the

same strain as a control, and this does provide a reasonable starting point for studies.

However, as mechanisms of virulence and attenuation can very between strains and

hosts, determination of 50% infection or lethal doses along with bacterial counts would

give a much better indication of differential virulence as illustrated in a more recent

study from the same group (Morris et al., Emerg Infect Dis. 2015 Jan;21(1):58-63). In the

absence of basic histology/organ counts the authors should acknowledge that bacterial

colonization, dissemination, tissue tropism and other factors that lead to illness or death

may considerably between strains and could reflect different mechanisms that result in

the data observed. For example, attenuation in some cases may still result in

colonization…in other cases this may not be the case. Furthermore, if the dose for

inoculation is well below an ID/LD50 one must be cautious in describing the observed

results as translation to differential virulence or even attenuation in humans as strains

that did not result in death in this study could still be very lethal. This is a reason why

high doses of organisms are used in protection studies as it is not uncommon to see all

mice survive if the inoculation dose is not titrated correctly. And also why it is essential

to minimally characterize bacterial counts in all animals at the end of the study.

In the absence of titration data for ID/LD50 values, bacterial counts and/or histology,

there is simply not enough evidence to support the description of the results as

indicative of differential virulence – where virulence is generally understood as the

capacity to cause disease in a host. What this study does support is that the strains

studied may indeed have different levels of infectious doses that results in different

lethality numbers over the period of the animal study – and this is worth publishing, The

authors could potentially argue that their data may suggest that differences in infectious

doses could result in variations of disease presentation in humans (but not necessarily a

lack of lethality) – though the amount of organism required to cause disease in humans

is also highly dependent on risk factors (age, diabetes, and other conditions that can

result in being immunocompromised).

**Response**: We agree that these results are suggestive of differential virulence, but not definitive. We have added additional language to the text to make this point clear, highlighting assumptions, including unknown questions of colonization, and putting our results in context.

2. Machine Learning

With all due respect to the authors, this reviewer is struggling to understand exactly

what input were used to undertake the ML predictions. The methodology indicates a

“LS-BSR matrix” but no other information is provided so it is difficult to know how the

ML is being used here to identify patterns/correlations.

**Response:** We have added more detail to explain how the BSR values for coding regions was converted to a binary matrix based on defined thresholds. This is common practice in other GWAS methods (e.g. Scoary).

How is the random forest classifier being used in this model? Are there gaps in the data? I understand that its use will reduce overfitting but what is the training set that being used? Is the LS-BSR matrix generated exclusively using sequences described in this manuscript? If so, then it is possible that the results generated are heavily biased and this may explain my concerns

about the identification of sequence variation in Hcp1 as being linked to observed

results from the animal model studies.

**Response**: The random forest hyperparameters were tuned to generate an optimal model to predict virulence on the dataset described. From there, the tuned random forest model is used as the backbone within the Boruta algorithm to calculate feature importance, thereby determining which features are selected. We have added text to the revised manuscript to highlight that Boruta was run on the tuned model. We don’t understand the question on gaps in the data; if this is in reference to missing data, then no, we don’t include any missing data in our model. Only data generated in this study was used for the model, as we don’t know the phenotype of genomes in other studies. Even if the virulence phenotype is known from external sequences, the virulence model may be different, which makes comparison difficult. We have added additional language to the text stating our use of stratified 5-fold cross-validation, which is how we reduced overfitting when tuning the random forest hyperparameters.

Indeed, the authors themselves note that the ML Boruta model has not been previously

used for bacterial GWAS and this reviewer suspects there may be a reason for this.

**Response**: We cite relevant published studies that have used this method. The method performs robust re-sampling to limit the number of false-positive associations. If there is sound logic for why the method was not appropriate for the analyses performed here, then it should be specifically raised by the reviewer.

These authors were previously asked to consider benchmarking the use of this method

using established sequence data that is known to impact on the virulence properties of

B. pseudomallei. The justification provided for using this ML approach “to identify genes

associated with virulence that were not obvious by other methods” is a weak statement

as the meaning of “not obvious” needs to be clearly defined by the authors using

concrete evidence from their data analyses that led to this decision. My concern is that

in absence of benchmarking, that the correlations shown in Figure 6 may be very weak

or artefacts.

**Response**: Many of the regions found in Figure 6 were supported by orthogonal methods, suggesting that the associations are not weak. The Boruta algorithm, through its use of shadow feature re-sampling, should reduce the number of false-positive associations. If we had run a chi-square analyses, which is used by other GWAS methods, the number of possible associations would have been large, although most likely not biologically significant. The authors agree that the use of “not obvious” was not clear and has been removed.

ML can be very useful, and I am quite supportive of its application as an exploratory

method, but the results obtained require more analysis then just providing a list of

sequences on a novel dataset to support the use of a previously untested methodology

in analysis of bacterial sequences. If the authors still wish to publish these data then

there needs to be more discussion about the risks of the approach and the need for

benchmarking and better validation.

**Response**: The ML results were used to down select potential associations and were largely supportive of results obtained by other methods. We already put a caveat on the ML results, but we’ve now added text suggesting that benchmarking studies are required for full validation.

3. Hcp1 sequence analysis and interpretation

The authors make the following statements in this manuscript:“However, 14 of 20 attenuated isolates, plus one intermediate virulence isolate (Bp8881), contain a variant of Hcp1 (C1W89_RS19320) with significant amino acid differences to BPSS1498 (Figure S7). This demonstrates that this Hcp1 homolog has a different peptide composition and may have a different protein function associated with attenuated virulence.”

“Of external ST93 genome assemblies, 3 have the K96243 version of Hcp1, while 5 have

the alternate allele; one of the external genomes with the alternate Hcp1 allele was

associated with humans (SAMN04208633), although details of the isolation source are

not available in the public record.”

And in their reviewer responses they also state:

“We have changed the language to reflect that the change may be altered and not very

different. MUSCLE was used to align amino acid sequences and the citation and version

are in the original manuscript. There are no statistics, as even a single amino acid

difference would constitute a different protein sequence. There is a wealth of literature

in B. pseudomallei and other bacterial and viral pathogens to demonstrate that a single

amino acid difference can drastically affect a phenotype, which was the point that we

were making.”

It is evident from the results obtained in the animal model studies and that the authors

are trying to find an explanation for the observed results using sequence analysis

techniques and this is understandable. However, this reviewer does not support the

interpretation of the Hcp1 variant and considers it to be flawed for the following

reasons:

1. Although the authors’ justification for suggesting that the Hcp1 sequence variant

may have a different function based on its composition, this is an extreme

interpretation compared to prevailing knowledge about protein-structure function

relationships, protein evolution and the identification of homologs within and across

species boundaries. For example, within a single bacterial species, full genome

sequencing of different strains and subsequent translation of encoded gene products

demonstrate single (or more) residue changes in the majority of encoded proteins

without a loss or gain of function.

**Response:** We have tried to make the argument that the association is suggestive but requires experimental validation studies. We have changed the language in the text to suggest that the amino acid differences may result in different protein activity, rather than function. Even that statement is speculative and of course will require experimental validation.

2. The bioinformatic analyses undertaken is incomplete and lacks rigor. A standard and

basic analysis of the variant sequence using the BlastP suite (any of the options)

against the non-redundant protein sequence database shows that this sequence

aligns with at least 100 annotated sequences for Hcp1/Hcp Type IV effector family

protein with many bacterial species – with tops hits being B. pseudomallei.

**Response**: We don’t understand the analysis that was performed by the reviewer. The fact that the protein sequence is associated with B. pseudomallei is not surprising and consistent with our findings. We acknowledge that multiple protein variants of Hcp1 may exist, but why broaden the analyses to beyond the strains compared in this study? The analysis reported in this study was a comparison between two different alleles of Hcp1.

The alignment with the Hcp1 sequence is shown below. It has an identity of 98% with three

amino acid changes – a conservative substitution and an inversion. A skilled protein

bioinformaticist would not consider these changes likely to lead to any gain or loss of

function.

**Response**: We don’t see the alignment in our review document and we’re unsure if the reviewer is trying to replicate our results. In our analysis, we demonstrate many differences between protein sequences observed in our dataset (shown in Figure S7). This is the comparison that is relevant in our study.

4. The unlikeliness of this sequence functioning in any way other than as annotated as

Hcp1 is further supported by the identification of the identical sequence in 376

entries in the NCBI (see below). The strains listed have shown this sequence to be

identified in isolates from clinical strains and environmental samples, some of which

contain information about the location and source of isolation.

**Response:** We agree that the protein function is likely to be similar, assuming that the variant is functional, which we don’t know. We know from Bp that single gene differences do not always result in differential virulence across diverse backgrounds. Therefore, the identical protein in clinical and environmental isolates, especially amongst diverse genomic backgrounds, does not mean that the gene is not associated with virulence. For example, we published a paper showing complete attenuation in patient strains that contained all known virulence genes (https://journals.plos.org/plospathogens/article?id=10.1371/journal.ppat.1008298).

Given the wealth of information available (and this analysis could be further extended if

needed), the case for suggesting that this Hcp1 variant sequence “has a different peptide

composition and may have a different protein function associated with attenuated

virulence” – is not adequately made – taking into account the animal experiments and high

sequence identity of this variant to a nearly identical sequence present in many virulent B.

pseudomallei strains. Choosing to use MUSCLE to align the sequence with the sequence

from Hcp1 sequence from the K96243 strain gives a biased and incomplete analysis given

the large amount of sequencing data available and the ease with which to conduct searches

usually publicly available software that is considered reliable by most of the molecular

biology community.

**Response**: The goal of this study was not to perform a global analysis of the genomics of B. pseudomallei, although we have performed such studies, but to focus on the isolates from a single hole. Our methods in this paper and others go beyond blast searches in the ncbi portal, which ignores population structure, and are vital for the contextualization of these results. Protein bioinformaticists typically carry out alignments and searches using several different software to take into account different underlying biasing in alignment algorithms (e.g. global vs. local alignment).

Finally, the suggestion that there is a wealth of literature in B.

pseudomallei and other pathogens to demonstrate that a single amino acid change can

change a phenotype is true. However, the virulence of B. pseudomallei has always been

considered multi-factorial (not generally reliant upon a single factor like a toxin). It would be

an interesting analysis to cite the published studies of naturally occurring strains of B.

pseudomallei where a single residue change has been directly ascribed to a change in

phenotype (other than a truncation of loss of protein) and to include those references in the

discussion as they would be very valuable.

**Response**: Our published study of P314 describes naturally occurring strains of Bp that have acquired different phenotypes due to single mutations. That citation has now been added to the text, as suggested by the reviewer.

In summary, this reviewer does consider this paper worthy of publication but with a careful

re-analysis of the data and the methods used, and a more restrained interpretation of the

results.
